# Revealing two important tryptophan residues with completely different roles in a dye-decolorizing peroxidase from *Irpex lacteus* F17

**DOI:** 10.1186/s13068-021-01978-y

**Published:** 2021-05-31

**Authors:** Liuqing Li, Tao Wang, Taohua Chen, Wenhan Huang, Yinliang Zhang, Rong Jia, Chao He

**Affiliations:** 1 School of Life Science, Economic and Technology Development Zone, Anhui University, 111 jiulong Road, Hefei, Anhui PR China 230601; 2 Anhui Key Laboratory of Modern Biomanufacturing, Anhui University, Hefei, Anhui Province, China

**Keywords:** Dye-decolorizing peroxidase, *Irpex lacteus* F17, Crystal structure, Tryptophan residues, Substrates’ oxidation

## Abstract

**Background:**

Dye-decolorizing peroxidases (DyPs) represent a novel family of heme peroxidases that use H_2_O_2_ as the final electron acceptor to catalyze the oxidation of various organic compounds. A DyP from *Irpex lacteus* F17 (*Il*-DyP4, corresponding to GenBank MG209114), obtained by heterologous expression, exhibits a high catalytic efficiency for phenolic compounds and a strong decolorizing ability toward various synthetic dyes. However, the enzyme structure and the catalytic residues involved in substrate oxidation remain poorly understood.

**Results:**

Here, we obtained a high-resolution structure (2.0 Å, PDB: 7D8M) of *Il*‑DyP4 with α-helices, anti-parallel β-sheets and one ferric heme cofactor sandwiched between two domains. The crystal structure of *Il*‑DyP4 revealed two heme access channels leading from the enzyme molecular surface to its heme region, and also showed four conserved amino acid residues forming the pocket for the conversion of hydrogen peroxide into the water molecule. In addition, we found that Trp264 and Trp380, were two important residues with different roles in *Il*‑DyP4, by using site-directed mutagenesis and an electron paramagnetic resonance (EPR) study. Trp264 is a noncatalytic residue that mainly is used for maintaining the normal spatial conformation of the heme region and the high-spin state of heme Fe^3+^ of *Il*‑DyP4, while Trp380 serves as the surface-exposed radical-forming residue that is closely related to the oxidation of substrates including not only bulky dyes, but also simple phenols.

**Conclusions:**

This study is important for better understanding the catalytic properties of fungal DyPs and their structure–function relationships.

**Supplementary Information:**

The online version contains supplementary material available at 10.1186/s13068-021-01978-y.

## Background

Dye-decolorizing peroxidases (DyPs) comprise a new family of heme peroxidases, which are commonly present in microorganisms, including fungi, bacteria and archaea [1, 2]. The DyPs were first discovered in the fungus *Geotrichum candidum* Dec 1 (identified later as *Bjerkandera adusta*), which was shown to be capable of decoloring 18 kinds of reactive, acidic and dispersive dyes [3]. The first cloned DyP from *B. adusta* Dec 1 was composed of 498 amino acids (*M*r = 53,306) [4], and lacked the typical conserved motif that usually exists in plant peroxidases. Subsequently, more DyP-type proteins from bacteria and fungi were reported, including YcdB from *Escherichia coli*, *Bt*DyP from *Bacteroides thetaiotaomicron*, TyrA from *Shewanella oneidensis*, as well as DyPs from the basidiomycetes *Pleurotus ostreatus* and *Marasmius scorodonius* [5–9].

Based on sequence homology analyses, the DyPs family can be divided into four subfamilies (class A to D) in the peroxidase database, Peroxi-Base [10–12]. The genes encoding class A, B and C proteins are found in bacteria and lower eukaryotes, while genes encoding class D are found in fungi (mainly basidiomycetes). The four classes of proteins show a similar tertiary structure, while the catalytic abilities of these proteins are different. The class D DyPs possesses the highest oxidative activity among four subfamilies of DyPs [12]. In addition, the amino acid sequence identity between any two subfamilies is low. From a pairwise comparison, the class-D protein shows the lowest sequence identity with the other three classes (7%, 7% and 16%, respectively) [12, 13]. Therefore, according to the result from the structure-based sequence alignments across subfamilies, Yoshida and Sugano proposed a new classification system for the DyPs family. DyPs were reclassified into 3 classes: class P (primitive, former class B), class I (intermediate, former class A), and class V (advanced, former classes C and D) [12].

The biochemical properties of some fungal and bacterial DyPs have been characterized thus far [6, 7, 14–17], and the crystal structures of a few DyPs have also been solved, including *Bad*DyP of *Bjerkandera adusta* [18], *Aau*DyP of *Auricularia auricula-judae* [19], *Bt*DyP from *B. thetaiotaomicron* and TyrA from *S. oneidensis* [6, 7]. Unlike the crystal structures of fungal class II heme peroxidases, such as lignin peroxidases (LiPs; EC 1.11.1.14), manganese peroxidases (MnPs; EC 1.11.1.13) and versatile peroxidases (VPs; EC 1.11.1.16), DyPs exhibit a unique tertiary structure: (I) DyPs have two domains, composed of α-helix and β-sheet, to form the α/β barrel structure, which is vastly different from the α-helix-rich structure of fungal class II heme peroxidases [7, 20, 21]; (II) the heme molecule binds to the C-terminal region of DyPs protein. In addition, a conserved acidic amino acid (usually aspartic acid) was found in the heme distal region, which is also different from the distal conserved histidine in fungal class II heme peroxidases [2, 18, 22]. Moreover, DyPs show low sequence identity with LiPs and MnPs (0.5–5%), and have more potential radical-forming residues (the basidiomycetes DyPs have an average of 8.8 tyrosines and 5.7 tryptophans in their amino acid sequences) than the fungal class II heme peroxidases [16, 20, 23]. DyPs also contain another conserved motif, GXXDG, in contrast to the RXXF/WH motif (R and H represent the conserved arginine and histidine in the distal heme region, respectively) in fungal class II heme peroxidases [4, 21].

Despite the presence of many unique structural characteristics of DyPs, the catalytic cycle of DyPs is similar to that of other well-known heme peroxidases. First, hydrogen peroxide (H_2_O_2_) directly oxidizes the native ferric enzyme to produce compound I (Cpd I) containing Fe^4+^ = O and porphyrin cationic radical (Por^+^•). Then, Cpd I is reduced back to the native ferric enzyme via two successive single-electron reductions through the oxidation of the reducing substrates (such as phenols and dyes), and produced an intermediate compound II (Cpd II, [Fe^4+^ = O] Por) and the native ferric enzyme in turn [1, 2, 19]. However, unlike H_2_O_2_, some sterically bulky substrates, such as anthraquinone and azo dyes, are unable to gain access to the heme cavity of a DyP [19, 24], which means that the oxidation site in DyPs is more likely to localize on the enzyme surface. This site for the oxidation of bulky substrates is the substrate–intermediate–protein radical center on which electron transfer to the heme is initiated to complete the catalytic cycle of the enzyme. That is to say, the long-range electron transfer from radical-forming exposed tryptophan or tyrosine (usually tryptophan in most of the heme peroxidases) is suggested for the oxidation of large-size substrates by DyPs, just like LiPs and VPs, which have been found to oxidize substrates via surface-exposed tryptophanyl radicals [25–27].

*Irpex lacteus* F17 (China Center for Type Culture Collection: CCTCC AF 2,014,020) is a white-rot fungus that was isolated from a decaying hardwood tree and stored in our laboratory. In taxonomy, *I. lacteus* F17 resides in the Fungal Kingdom of the Eukaryota, and belongs to the family Polyporaceae, order Polyporales, class Basidiomycetes, Phylum Basidiomycota [28]. Previously, we performed extensive studies to demonstrate the strong ability to degrade recalcitrant aromatic pollutants by this strain and unique characteristics of MnP, which proved to be the main class II heme peroxidase produced by *I. lacteus* F17 [29–31]. Recently, we characterized the genome of this strain and found it has 14 class II heme peroxidases (one LiP and 13 MnPs) and five DyPs [28]. Among the five *I. lacteus* DyPs (*Il*-DyP1-5), *Il*‑DyP4 is the first recombinant enzyme obtained by heterologous expression via *E. coli*, and was well characterized by biochemical and electrochemical studies [32]. Additionally, compared with an MnP (*Il*-MnP6) from *I. lacteus* F17, *Il*‑DyP4 showed a strong ability to decolorize different types of dyes, including anthraquinone dyes, azo dyes, phenazine dyes, triphenylmethane dyes, as well as aniline dyes.

As mentioned above, *Il*‑DyP4 exhibits the potential environmental applications. However, its crystal structure, catalytic site(s), as well as the mode for substrate oxidation remain elusive. Here, we report the crystal structure of DyP from *I. lacteus*. We found two important tryptophan residues in *Il*‑DyP4 with completely different functions using site-directed mutagenesis and electron paramagnetic resonance (EPR) measurements. This study provides insights into the catalytic action of DyPs and contributes to their molecular modification for further improving the value of industrial applications.

## Methods

### Heterologous expression, in vitro refolding and purification of *Il‑*DyP4

The recombinant vector pET28a-*Il*-DyP4 was constructed according to the method of Duan et al. [32]. The positive transformant (*E. coli* Rosetta) harboring pET28a-*Il*-DyP4 was cultured at 37 °C in Luria broth medium (400 mL) containing kanamycin and chloramphenicol; isopropyl-β-d-thiogalactoside was added to a final concentration of 0.5 mM for induction. The inclusion bodies containing recombinant *Il*‑DyP4 were obtained via cell disruption.

The inclusion bodies were solubilized in 5 mL of 50 mM Tris–HCl (pH 8.0) containing 8 M urea, 1 mM EDTA, for 3 h at 4 °C for the complete solubilization of the *Il*-DyP4 polypeptide. Then the optimal solution was used to refold the inactive polypeptide. The refolding conditions for the in vitro activation of *Il*-DyP4 contained 1 mM EDTA, 5 μM hemin, and 0.75 M urea in 10 mM sodium acetate buffer (pH 6.0) at 4 °C for 36 h as previously described [32]. After the incubation of 36 h, the refolded solutions containing recombinant *Il*-DyP4 were dialysed in 10 mM sodium acetate buffer (pH 6.0, 10 times volume of the refolded solutions) at 4 °C for 24 h, then the solution was centrifuged (12,000 rpm) at 4 °C for 20 min to remove any insoluble protein and excess hemin. Furthermore, above protein solutions were purified using Ni–NTA affinity chromatography (Sangon Biotech, Shanghai, China) according to the manufacturer’s instructions. After that, the fractions containing *Il*-DyP4 were subjected to several cycles of concentration and solvent exchange with sodium acetate buffer (pH 6.0), carried out at 4 °C in Amicon 10-kDa centrifugal filters. Then sodium dodecyl sulfate polyacrylamide gel electrophoresis (SDS-PAGE) was performed and stained with Coomassie Brilliant Blue R-250 for confirming the purity of the enzymes. Protein content was measured using the Bradford method with bovine serum albumin as the standard. The specific activity of *Il*‑DyP4 was calculated based upon U mg^–1^ of protein per mL of enzyme solution and one unit (U) was defined as the amount of enzyme that oxidized 1 μmol of substrate per minute.

### Crystallization and structure determination of *Il*‑DyP4

Before crystallization, the *Il*‑DyP4 solutions purified by Ni–NTA affinity chromatography was further purified using a fast protein liquid chromatography system with Superdex 75 column (GE healthcare) and concentrated to 12 mg/mL of protein solution. The Reinheitszahl value (Rz) of the purified *Il*‑DyP4 was found to be about 2.0 according to the ratio between absorbance at the wavelength of the Soret band and the absorbance at 280 nm (A405/A280). According to the Lambert–Beer equation, the molar extinction coefficient of *Il*‑DyP4 at 405 nm was estimated as 222,196 M^−1^ cm^−1^ in 10 mM sodium acetate buffer (pH 4.5), from a Bradford determination of pure protein concentration. Crystals were grown by the sitting drop vapor diffusion method in a solution (pH 5.2) consisting of 0.2 M ammonium acetate, 0.1 M sodium acetate trihydrate and 34% (w/v) PEG 4000 at 16 ℃ for 6 days, then stored in reservoir buffer containing 25% glycerol as cryoprotectant and quickly frozen in liquid nitrogen for X-ray diffraction at Shanghai Synchrotron Radiation Facility (Shanghai, China). Diffraction data were indexed, integrated and scaled using HKL2000. The structure was solved by molecular replacement using the crystal structure of *Bad*DyP (PDB: 3AFV) as the search model using the program Molrep. An initial model was built with ARP/wARP, and the final model was obtained by successive refinement using COOT and Refmac5.

### Chemical modification and site-directed mutagenesis of *Il*-DyP4

Chemical modification was used for estimating the effect of tryptophan residues in *Il*-DyP4. Tryptophan residues in 0.2 μM recombinant *Il*‑DyP4 were chemically modified using up to 5.0 mM N-bromosuccinimide (NBS) at 25 ℃ for 30 min, then the residual oxidative activity and decolorizing ability of the chemically modified enzymes were measured as described below (characterization of the oxidative ability of Il-DyP4 and the variants).

Site-directed mutagenesis with PCR using the QuikChange™ method was used for identifying the essential tryptophan residues in *Il*-DyP4. The plasmid pET28a-*Il*-DyP4 was used as a template and the primers are shown in Tabale S1. PCR reaction mixtures (50 μL final volume) contained 1 ng/μL template DNA, 0.5 mmol/L dNTPs, 10 μmol/L direct and reverse primers, 0.5 μL PrimeSTAR HS Polymerase. Reaction conditions were as follows: (i) a “hotstart” at 94 °C for 10 min; (ii) 25 cycles at 98 °C for 10 s, 55 °C for 5 s and 72 °C for 7 min; (iii) a final cycle at 72 °C for 10 min. The plasmid sequencing in individual clones was used in verifying the mutation, then *Il*‑DyP4 variants were expressed, refolded and purified by the same methods used in the production of recombinant *Il*‑DyP4 (see heterologous expression, in vitro refolding and purification of *Il*‑DyP4). The heme content of *Il*‑DyP4 variants was determined by the pyridine ferro-hemochrome method using an extinction coefficient of pyridine hemochrome (εR-O) of 28.32 mM^−1^ cm^−1^ at 556 nm [22]. Pyridine hemochrome of each variant was measured using 1.303 μM of protein concentration, which was calculated from the molar extinction coefficient of *Il*‑DyP4 (ε405 222,196 M^−1^ cm^−1^).

### Characterization of the oxidative ability of *Il*-DyP4 and the variants

The substrates used in this assay were 2,6-dimethoxyphenol (the extinction coefficient of the DMP oxidation product is ε_469_ = 49,600 M^−1^ cm^−1^), guaiacol (the extinction coefficient of the guaiacol oxidation product is *ε*_456_ = 12,100 M^−1^ cm^−1^), 2,2’-azino-bis(3-ethylbenzthiazoline-6-sulfonic acid) (the extinction coefficient of the ABTS oxidation product is *ε*_420_ = 36,000 M^−1^ cm^−1^); reactive blue 4 (RB 4, *λ*_max_ = 598 nm), reactive blue 5 (RB 5, *λ*_max_ = 600 nm), reactive blue 19 (RB 19, *λ*_max_ = 595 nm), direct sky blue 5B (5B, *λ*_max_ = 598 nm), methyl orange (MO, λ_max_ = 464 nm) and reactive violet 5 (RV 5, *λ*_max_ = 570 nm). The chemical structures of above substrates are shown in Additional file 1: Table S2.

The oxidative activity for simple substrates (DMP, guaiacol and ABTS) was estimated spectrophotometrically by the oxidation of 1 mM substrates in 10 mM sodium tartrate buffer (pH 3.5) at 35 °C. The reaction was initiated by 0.1 mM H_2_O_2_, samples without enzymes served as the control. The decolorizing rates for bulky dyes, including anthraquinone dyes (RB 4, RB 5 and RB 19) and azo dyes (5B, MO and RV 5) were measured in the assay mixtures (1 mL) containing enzyme (0.5 μg/mL), dye (25–200 μM) and H_2_O_2_ (0.1 mM). The reactions lasted for 3 min in 10 mM sodium tartrate buffer (pH 3.5) at 35 °C. Samples without enzymes served as the control.

The decolorization percentage was calculated according to below equation (Eq. 1):

1 $$\rm{Decolorizing rate} \left( \% \right){\text{ }} = {\text{ }}\left[ {\left( {{A_0} - {\text{ }}{A_t}} \right)/{A_0}} \right]{\text{ }} \times {\text{ }}100\%$$

where *A*_0_ is the initial absorbance at λ_max_ and A_t_ refers to the absorbance at *λ*_max_ at reaction time t.

### Measurement of steady-state kinetic constants of enzymes

The steady-state kinetic constants were determined by incubating the enzyme (~ 3.3 nM) in 10 mM sodium tartrate buffer (pH 3.5) with a set concentration of a reducing substrate. ABTS, DMP and RB19 (the extinction coefficient of the RB19 oxidation product is *ε*_595_ = 10,000 M^−1^ cm^−1^) were used as substrates for the assays. All of the kinetic constants measurements were performed at 3.3 nM of enzyme concentration, which was determined spectrophotometrically using the molar extinction coefficient of *Il*‑DyP4 (*ε*405 222,196 M^−1^ cm^−1^). The kinetic constants were calculated from absorbance changes during substrate oxidation in the presence of 0.1 mM H_2_O_2_ using a UV–visible spectrophotometer (Shanghai, China). The reaction was initiated by 0.1 mM H_2_O_2_, samples without enzymes served as the control. In addition, the steady-state kinetic constants for H_2_O_2_ substrate were also estimated spectrophotometrically by the oxidation of 1 mM ABTS in 10 mM sodium tartrate buffer (pH 3.5) at 35 °C. The reaction was initiated by H_2_O_2_ with increasing H_2_O_2_ concentrations, samples without enzymes served as the control.

Through the hyperbolic, non-linear least square method, the Michaelis constants (*K*_*m*_), catalytic constants (*k*_*cat*_) and rate constants (*k*_*cat*_/*K*_*m*_) were obtained by the Michaelis–Menten equation (Eqs. 2 and  3):

2 $${\text{v }} = {\text{ }}{V_{max}}\left[ S \right]/\left( {{K_m} + \left[ S \right]} \right)$$

3 $${k_{cat}} = {\text{ }}{V_{max}}/{E_{total\;}}$$

where v is the measured initial velocity of the enzymatic reaction, and *V*_max_ is the maximum reaction rate; [S] is the substrate concentration, and *E*_total_ is the total enzyme concentration of reaction system.

### Spectral characterization of *Il*‑DyP4 and the variants

The UV–vis spectra were obtained using a DU 730 UV spectrophotometer in the 250–700 nm region. The fluorescence spectra were obtained at an excitation wavelength of 295 nm using a cuvette with a 10-mm path length. Excitation and emission bandwidths were set at 5 nm. All samples used in these assays contained about 0.1–0.11 mg/mL of each of the *Il*‑DyP4 variants in 10 mM sodium acetate buffer, pH 6.0. The concentration of the variants was calculated using the molar absorbance coefficient of *Il*‑DyP4 (*ε*_405 nm_ 222,196 M^−1^ cm^−1^).

### Electron paramagnetic resonance spectroscopy analysis

For analyzing the spin state of the heme iron and the protein-based radical signal, low temperature (10 K) 9-GHz EPR spectroscopy analysis was performed with a Bruker EMX plus 10/12 equipped with Oxford ESR910 Liquid Helium cryostat (High Magnetic Field Laboratory, Hefei, China). Enzyme solutions consisting of 40 μM *Il*‑DyP4 and various variants in 10 mM sodium acetate buffer (pH 6.0) were used for the assays, respectively. The enzyme solutions were used directly for spin-state analysis of Fe^3+^, while tenfold excess H_2_O_2_ was added for the analysis of the protein-based radical signal. The addition of H_2_O_2_ was done directly in the EPR tube and the reaction time before freezing in liquid nitrogen was ~ 10 s.

## Results

### Crystallographic structural characterization

**Fig. 1:**
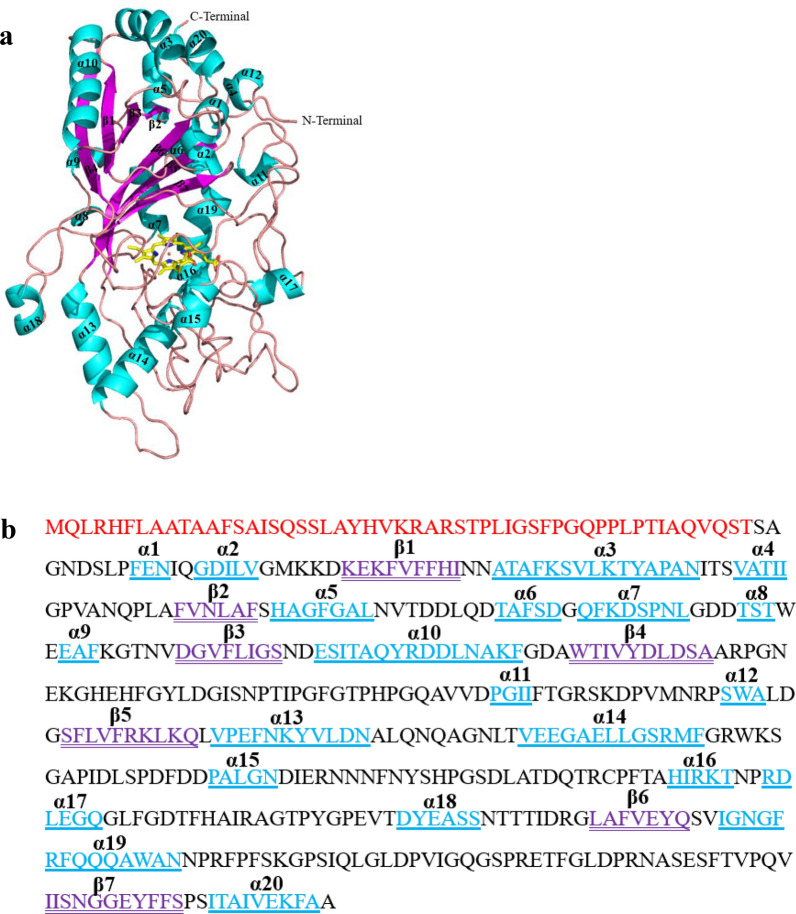
Crystal structure of *Il*-DyP4. **a** Ribbon model of *Il*-DyP4. Helices (α1–α20) were shown in cyan and β-strands (β1–β7) were shown in violet. **b** Diagram showing the secondary structural elements superimposed on primary sequence. The N-terminal signal peptide sequence prior to the mature protein was colored red

**Fig. 2:**
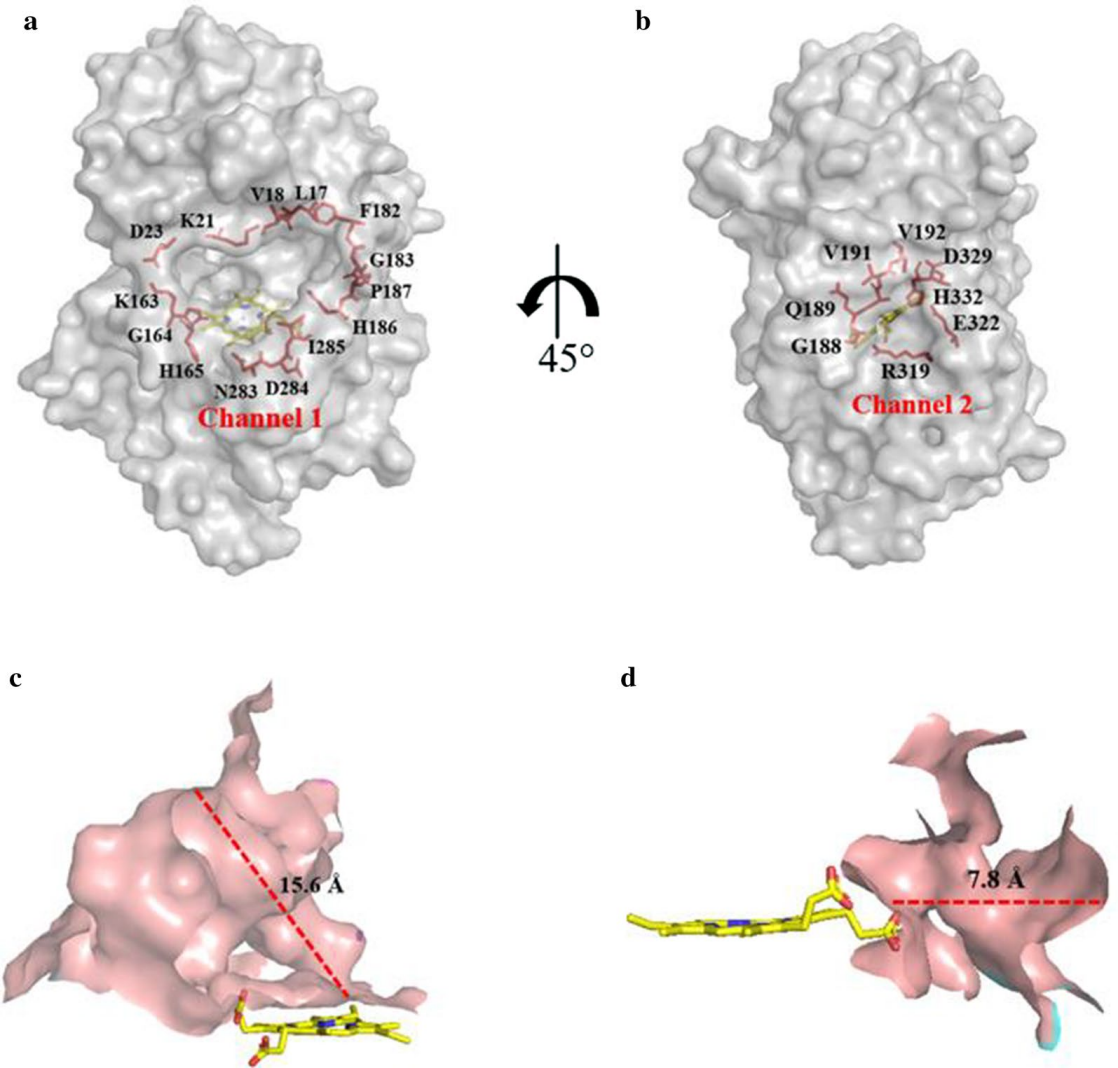
Two heme access channels in *Il*-DyP4 showing the surrounding residues and the length of channels. **a** Channel 1 and the surrounding residues at the protein surface entrance. **b** Channel 2 and the surrounding residues at the protein surface entrance. **c** The length of channel 1. **d** The length of channel 2. The yellow sticks represent the heme cofactor

The crystal structure of *Il*‑DyP4 with a resolution of 2.0 Å was obtained in the present study, and the X-ray data collection and refinement statistics are summarized in Table 1. The final model of *Il*‑DyP4 includes one protein monomer (residues 6–450), one heme molecule, one oxygen molecule and 431 water molecules. As shown in Fig. 1a, the overall structure of *Il*‑DyP4 was an irregular ellipsoidal-shape with dimensions of about 71 × 50 × 43 Å^3^. In more detail, *Il*‑DyP4 possessed 20 α-helices and 7 β-strands in total. As shown in Fig. 1a, b, α-helices are widely spread on the protein surface, while β-strands are located in the interior of protein to form two domains. One heme with an iron is located between the two domains, namely the proximal N- and the distal C-terminal domain. One unique motif was a ferredoxin-like fold, which is constructed by a four-stranded anti-parallel β-sheet (β1 and β4, β2 and β3) and two α-helices (α3 and α10). Additionally, two heme access channels are present in *Il*‑DyP4. As shown in Fig. 2a, c, channel 1 has a length of 15.6 Å, and is located over the heme plane, which is the main channel leading from the protein surface to the heme distal cavity. Channel 1 presents as funnel-shaped with one upper entrance (about 24.2 Å × 17.4 Å) constructed by 14 residues and one narrow bottom hole (about 4.0 Å × 4.0 Å) at the vicinity of the heme (Fig. 2a). Another heme access-channel, namely channel 2, from the protein surface leads to the heme propionate with a length of about 7.8 Å (Fig. 2b, d). Channel 2 is cylindric in shape, and there are about eight residues (Gly188, Gln189, Val191, Val192, Asp329, H332, Glu322 and Arg319) together to construct the protein surface entrance of channel 2 (Fig. 2b).

**Table 1:** Summary of data collection and refinement statistics for *Il*‑DyP4 (PDB: 7D8M)

	*Il*‑DyP4
Data collection	
Wavelength (Å)	0.9792
Space group	*P*6_5_
Cell parameters	
a, b, c (Å)	118.96, 118.96, 65.35
α, β, γ (°)	90, 90, 120
Resolution (Å)	40.00–2.00 (2.03–2.00)^a^
*R*_merge_ (%)	12.5 (81.5)
*CC*_1/2_	0.993 (0.927)
*I*/σ*I*	14.3 (1.67)
Completeness (%)	99.8 (99.7)
Average redundancy	10.1 (7.9)
Refinement statistics	
No. reflections (overall)	35,658
No. reflections (test set)	1812
*R*_work_/*R*_free_(%)	16.89/20.87
Number of atoms	
Protein	3487
HEM/O_2_	43/2
H_2_O	312
*B* factors (Å^2^)	
Protein	32.26
HEM/O_2_	24.63/35.74
H_2_O	36.48
r.m.s. deviations	
Bond lengths (Å)	0.006
Bond angles (°)	0.812
Rampage plot % residues	
Favored	98.00
Allowed	2.00
Outliers	0.00

^a^ Values in parentheses are for the highest-resolution shell

**Fig. 3:**
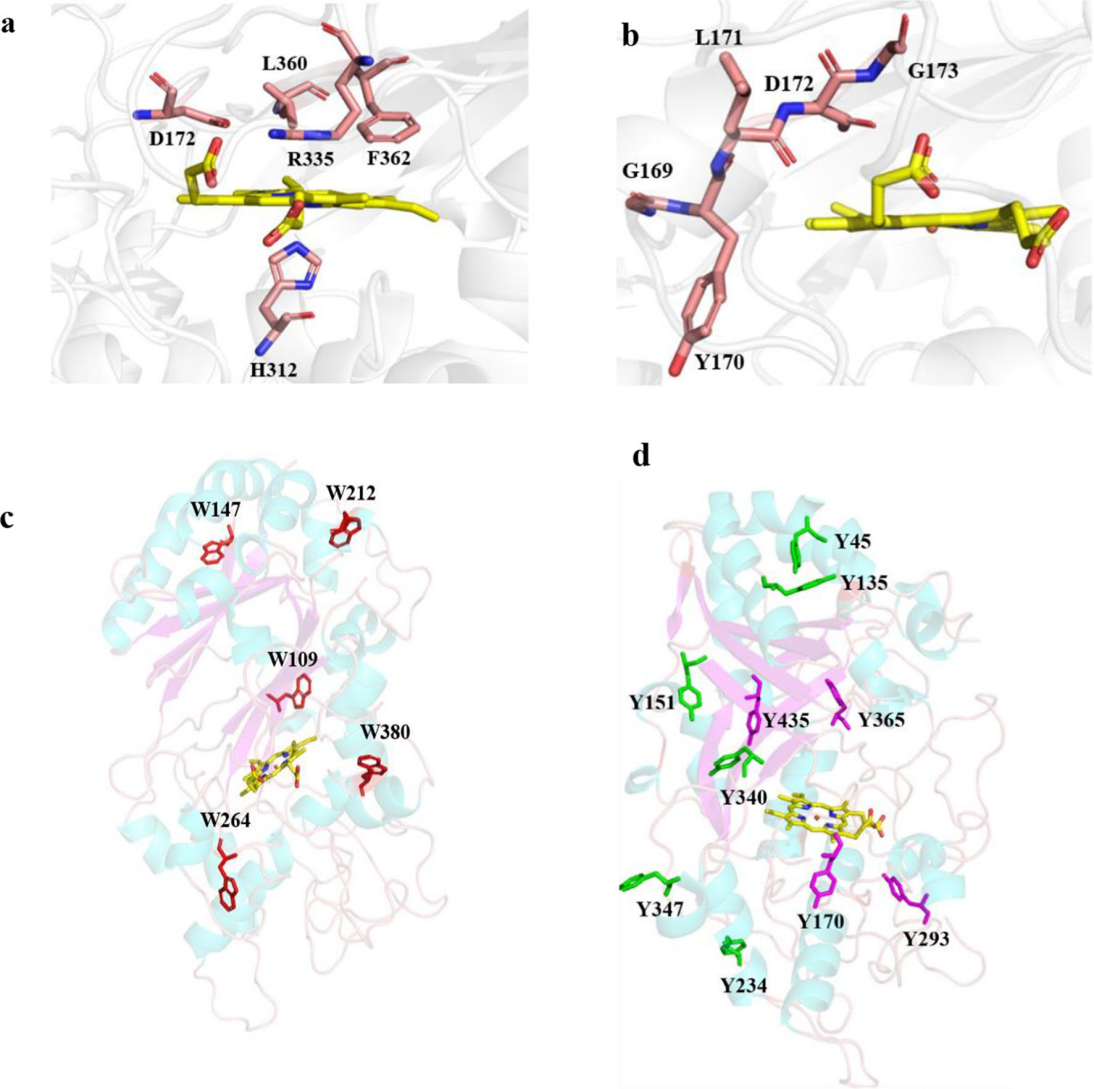
Important residues around the heme pocket and redox-active residues in *Il*-DyP4. **a** Distal pocket for the conversion of H_2_O_2_ into H_2_O and proximal heme iron ligand (His312). **b** Conserved GXXDG motif. **c**, **d** Tryptophan and tyrosine residues potentially involved in substrate oxidation at the protein surface. The red sticks and green sticks, respectively, represent the tryptophan and tyrosine residues located at the surface of *Il*-DyP4, while the purple sticks represent the tyrosine residues located in the interior of *Il*-DyP4 and yellow sticks represent the heme cofactor

In the heme active center, the side chains of Asp172, Arg335, Leu360 and Phe362 constructed a pocket structure which allows the conversion of H_2_O_2_ into H_2_O in the distal region of *Il*‑DyP4 (Fig. 3a). It is worth mentioning that Asp172 replaced the distal histidine that locates in the other heme peroxidases, which is consistent with the previous description of DyPs [33]. Meanwhile, *Il*‑DyP4 also has a histidine (His312), coordinated to the heme iron as the fifth ligand at a distance of 2.2 Å, which is the same as another heme peroxidases [34]. The conserved GXXDG motif in *Il*‑DyP4 is shown in Fig. 3b, which consisted of Gly169, Tyr170, Leu171, Asp172 and Gly173. The tryptophan and tyrosine residues potentially involved in oxidation of surface substrates are shown in Fig. 3c, d, respectively. There are a total of five tryptophan residues (Trp109, Trp147, Trp212, Trp264 and Trp380) and ten tyrosine residues (Tyr45, Tyr135, Tyr151, Tyr170, Tyr234, Tyr293, Tyr340, Tyr347, Tyr365 and Tyr435) in *Il*‑DyP4, and except for Tyr170, Tyr293, Tyr365 and Tyr435, all tryptophan and tyrosine residues are exposed to solvent to some degree. In addition, sequence alignment revealed that Trp109, Trp212, Trp380 and Tyr435 of *Il*‑DyP4 are conserved among the representative class V DyPs (Additional file 1: Fig. S1).

### Residual activity of *Il*‑DyP4 after chemical modification

**Fig. 4:**
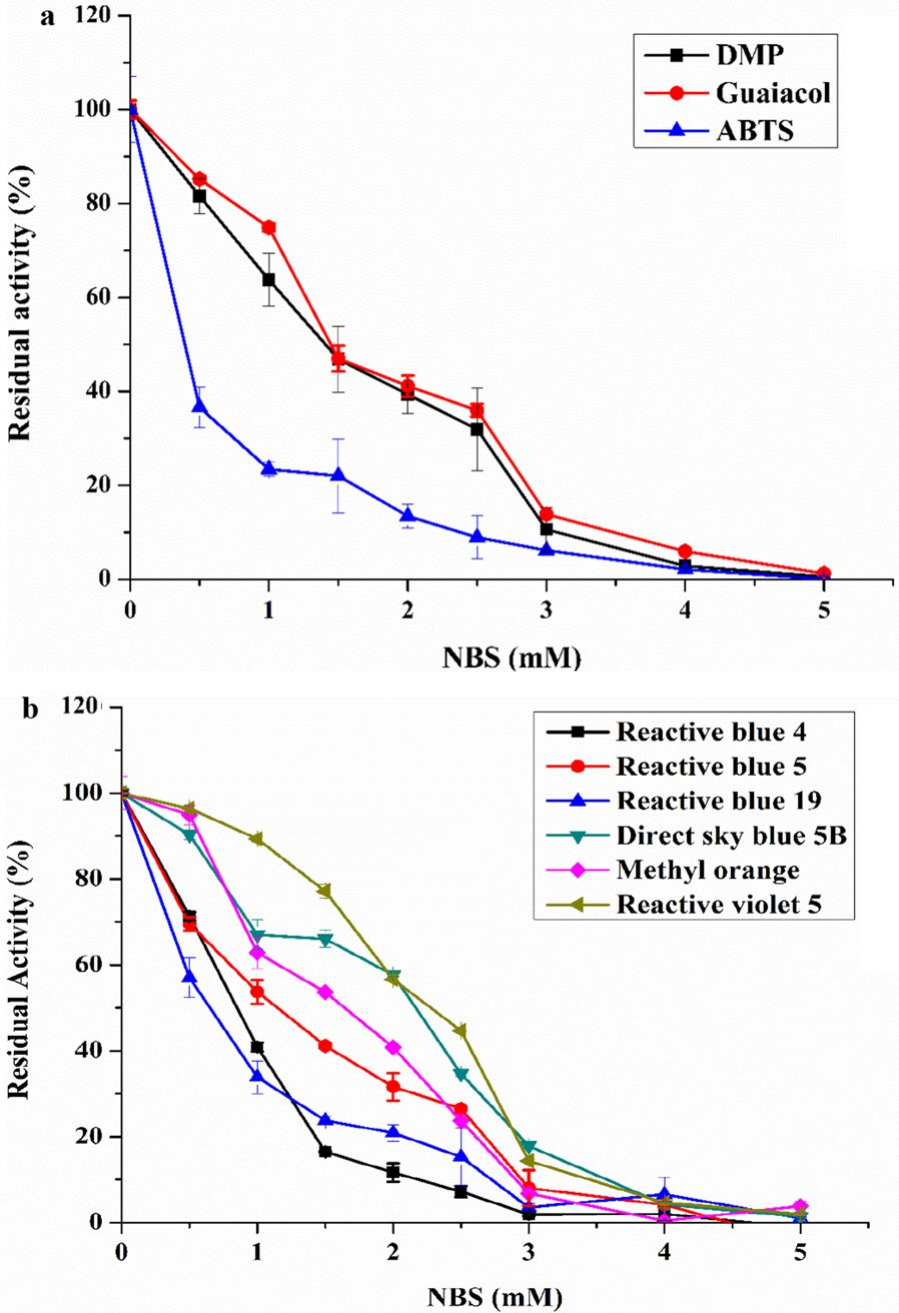
Residual activity of *Il*-DyP4 treated with increasing NBS concentrations for modification of Trp residues. Substrates: **a** Simple compounds, **b** Bulky dyes. The assay mixtures (1 mL) contained 1 mM of simple compounds or 25–200 μM of bulky dyes, 0.25 μg/mL (for simple compounds) or 0.5 μg/mL (for bulky dyes) of modified enzyme. The reaction was initiated by 0.1 mM H_2_O_2_ at 35 °C, pH 3.5. Samples without enzyme served as the control and the activity of untreated *Il*-DyP4 was taken as 100%

In this study, for the preliminary judgment of the importance of tryptophan residues in *Il*‑DyP4, chemical modification of tryptophan residues with NBS (oxidizes the tryptophan ring to oxindole) was carried out. As the results shown in Fig. 4, nearly 100% activity of *Il*‑DyP4 on the three simple-structured substrates, namely, DMP, guaiacol and ABTS (Fig. 4a) and the six bulk dyes, namely, RB 4, RB 5, RB 19, 5B, MO and RV 5 (Fig. 4b) were lost when NBS (up to 5 mM) was used. The results indicated that tryptophan residues are necessary for the catalytic activity of *Il*‑DyP4.

### Site-directed mutagenesis of the five tryptophan residues in *Il*‑Dyp4

#### Oxidative activity and decolorizing rate of the five W variants

**Fig. 5:**
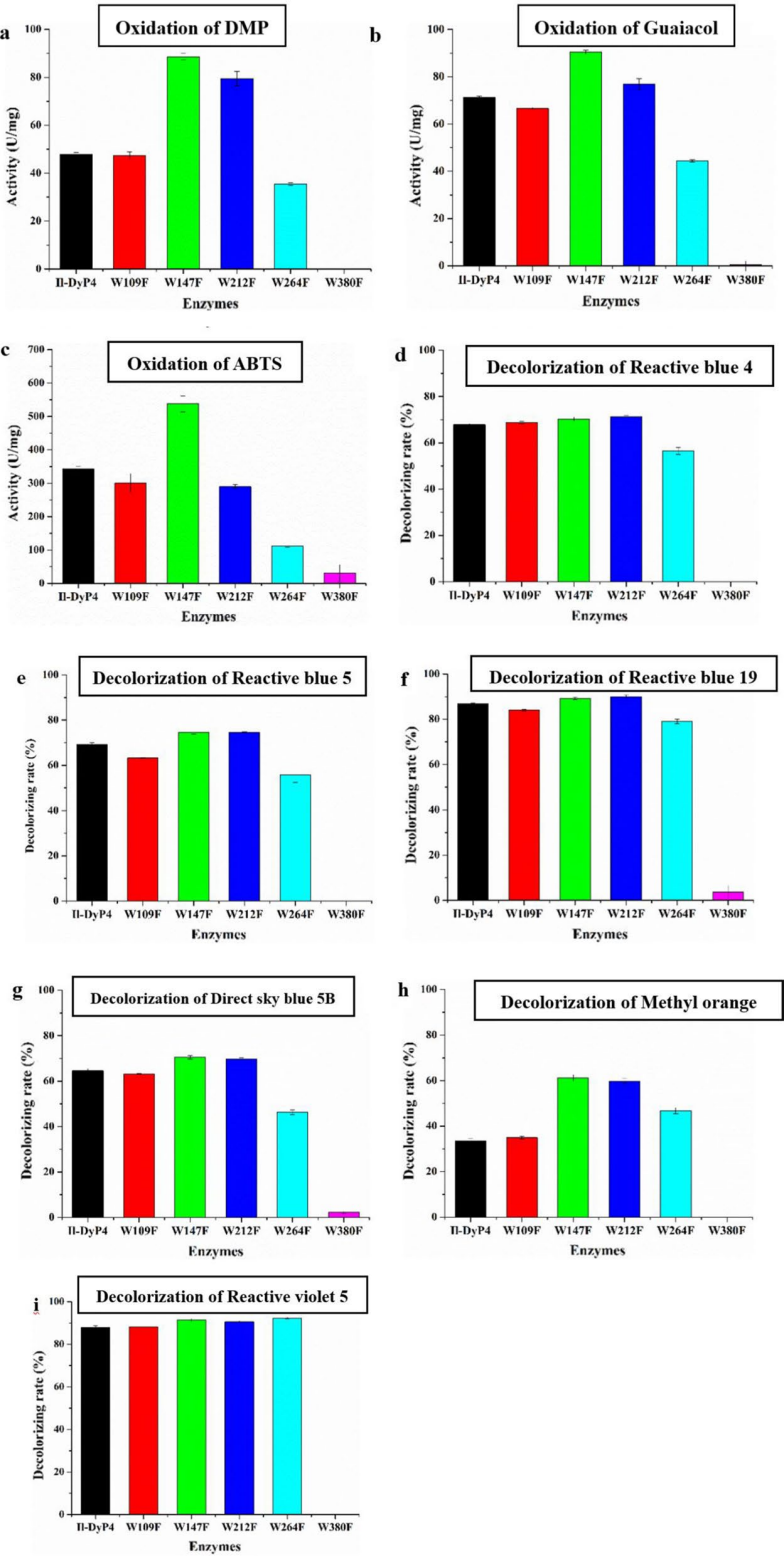
The oxidative activity and decolorizing rate of *Il*-DyP4 and the W variants. **a–c** The oxidative activity for DMP, guaiacol and ABTS, respectively. **d–i** The decolorizing rate for RB 4, RB 5, RB 19, 5B, MO and RV 5, respectively. The assay mixtures (1 mL) contained 1 mM of DMP, guaiacol and ABTS or 25–200 μM of dyes, 0.25 μg/mL (for oxidation assays) or 0.5 μg/mL (for decolorization assays) of purified enzyme. The reaction was initiated by 0.1 mM H_2_O_2_ at 35 °C, pH 3.5. Samples without enzyme served as the control

To determine the specific tryptophan residues that are associated with the *Il*‑DyP4 activity, all five tryptophan residues in *Il*‑DyP4 were substituted by phenylalanine (F) to obtain the W109F, W147F, W212F, W264F and W380F variants. The UV–vis spectra of these variants are shown in Additional file 1: Fig. S2a, and their heme *b* contents were about 0.79–0.97 mol/mol of protein using pyridine hemochromogen assays, similarly to the *Il*‑DyP4 (~ 1.01 mol/mol of protein). Then the oxidative activity for simple compounds (DMP, guaiacol and ABTS), as well as the decolorizing rate for bulk dyes (RB 4, RB 5, RB 19, 5B, MO and RV 5) were measured. As shown in Fig. 5, the oxidative activity and decolorizing rate of W264F were slightly lower than that of the other variants, except for W380F on the whole. The oxidative activity and decolorizing rate of W380F obviously decreased (almost 100% decolorizing ability was removed) compared with *Il*‑DyP4 and other variants. Thus, a decrease in oxidative ability was detected due to the replacement of W264 and W380 with a less oxidizable phenylalanine, respectively, indicating that W264 and W380 possessed a more important role than other tryptophan residues in *Il*‑DyP4.

#### Steady-state kinetic constants of Il ‑Dyp4 and the W variants

The steady-state kinetic constants of the five Trp variants were determined based on Michaelis–Menten equation (Eqs. 2 and 3). The results obtained from kinetic studies were consistent with the above oxidative and decolorizing assays. As seen in Table 2, the catalytic efficiency (*k*_*cat*_/*K*_*m*_) of W109F, W147F and W212F for ABTS, DMP and RB 19 had a similar order of magnitude compared with the *k*_*cat*_/*K*_*m*_ of *Il*‑DyP4 (10^7^, 10^6^ and 10^6^, respectively). The *k*_*cat*_/*K*_*m*_ of W264F for ABTS and DMP was found to be one order of magnitude lower than that of *Il*‑DyP4, while no difference in the *k*_*cat*_/*K*_*m*_ for RB 19 was observed. As for W380F, the *k*_*cat*_/*K*_*m*_ for ABTS was two orders of magnitude lower than that of *Il*‑DyP4 and the *k*_*cat*_/*K*_*m*_ for DMP and RB 19 were not calculated because the lack of enzymatic activity of W380F. Hence, it is clear that mutations of W380 to F380 had a more severe impact to the catalytic activity of *Il*‑DyP4 than the other four Trp variants, and mutations of W264 to F264 had a relatively little impact, compared with that of W380.

**Table 2:** Steady-state kinetic constants (*K*_*m*_ (μM), *k*_*cat*_ (s ^−1^), *k*_*cat*_/*K*_*m*_ (M^−1^ s^−1^)) for the oxidation of ABTS, DMP and RB 19 by *Il*-DyP4 and the W variants

	Kinetic constants^a^	*Il*-DyP4^c^	W109F^c^	W147F^c^	W212F^c^	W264F^c^	W380F^c^
ABTS	*K*_m_	67.29 + 13.05	66.44 ± 3.32	63.21 ± 12.86	71.48 ± 10.52	71.87 ± 20.86	752.00 ± 207.21
	*k*_cat_	790.00 ± 56.06	602.50 ± 11.00	786.70 ± 57.42	665.10 ± 36.90	384.85 ± 42.27	316.16 ± 61.61
	*k*_cat/_*K*_m_	(1.17 ± 0.43) × 10^7^	(9.03 ± 3.31) × 10^6^	(1.25 ± 0.45) × 10^7^	(9.30 ± 3.51) × 10^6^	(5.35 ± 2.02) × 10^6^	(4.20 ± 2.90) × 10^5^
DMP	*K*_m_	8.98 ± 1.02	172.71 ± 38.31	69.19 ± 10.56	86.67 ± 12.98	229.05 ± 30.79	–^b^
	*k*_cat_	58.99 ± 0.45	69.65 ± 3.46	72.66 ± 1.74	63.75 ± 1.62	71.21 ± 2.60	–^b^
	*k*_cat/_*K*_m_	(6.57 ± 0.44) × 10^6^	(4.03 ± 0.90) × 10^5^	(1.05 ± 0.16) × 10^6^	(7.3 ± 1.25) × 10^5^	(3.11 ± 0.84) × 10^5^	–^b^
RB 19	*K*_m_	139.87 ± 42.85	86.88 ± 12.28	97.59 ± 24.50	74.04 ± 14.38	27.60 ± 7.70	–^b^
	*k*_cat_	187.90 ± 37.00	112.63 ± 10.15	148.99 ± 21.92	108.79 ± 11.16	46.11 ± 5.91	–^b^
	*k*_cat/_*K*_m_	(1.34 ± 0.86) × 10^6^	(1.30 ± 0.83) × 10^6^	(1.53 ± 0.89) × 10^6^	(1.47 ± 0.78) × 10^6^	(1.67 ± 0.76) × 10^6^	–^b^

^a^All data were fitted using the hyperbola function by Origin

^b^Not calculated because of a lack of enzymatic activity

^c^Substrate oxidation was measured at pH 3.5 (10 mM sodium tartrate buffer) using 3.3 nM of enzyme concentration obtained from a molar extinction coefficient

### Investigation of the role of W264 in *Il*‑DyP4

#### Oxidative activity and decolorizing rate of the W264 variants

**Fig. 6:**
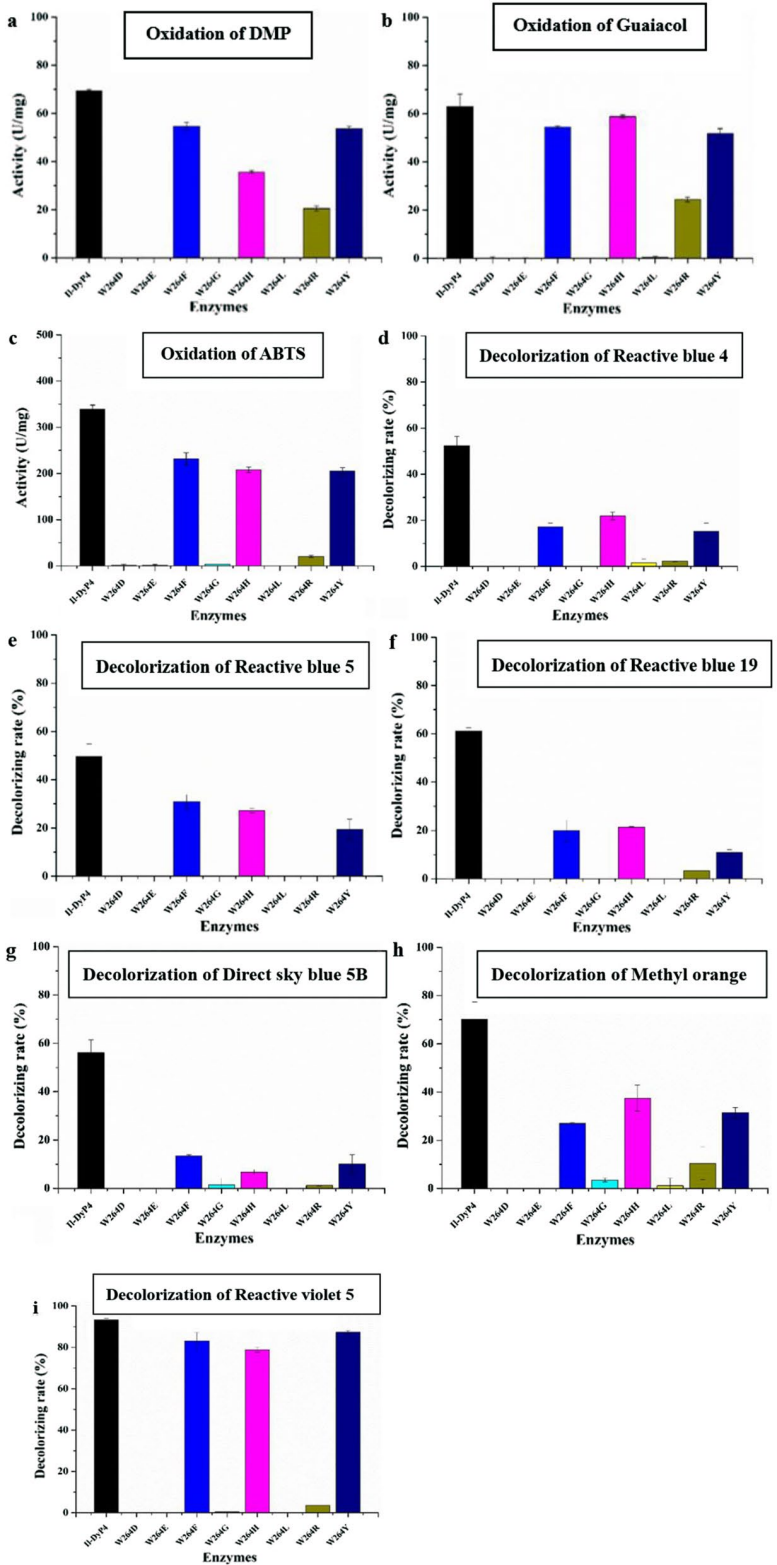
The oxidative activity and decolorizing rate of *Il*-DyP4 and the W264 variants. **a–c** The oxidative activity for DMP, guaiacol and ABTS, respectively. **d–i** The decolorizing rate for RB 4, RB 5, RB 19, 5B, MO and RV 5, respectively. The assay mixtures (1 mL) contained 1 mM of DMP, guaiacol and ABTS or 25–200 μM of dyes, 0.25 μg/mL (for oxidation assays) or 0.5 μg/mL (for decolorization assays) of purified enzyme. The reaction was initiated by 0.1 mM H_2_O_2_ at 35 °C, pH 3.5. Samples without enzyme served as the control

To investigate the role of W264, eight amino acids, namely, aspartic acid (D), glutamic acid (E), phenylalanine (F), glycine (G), histidine (H), leucine (L), arginine (R) and tyrosine (Y) were selected for the replacement of W264, respectively, by site-directed mutagenesis. These amino acids were chosen because they have a different polarity, hydrophobicity, and steric hindrance effect compared with the original W264. As shown in Fig. 6, the variants W264D, W264E, W264G, W264L and W264R lost almost all of their catalytic ability for DMP, guaiacol, ABTS and various dyes (with the exception that W264R retained ~ 40% oxidative activity for DMP and guaiacol) compared with *Il*‑DyP4. By contrast, the variants W264F, W264H and W264Y showed a stronger catalytic ability compared with the variants above, and had more than 70% of the *Il*‑DyP4 activity for DMP, guaiacol and ABTS oxidation, and more than 50% of the *Il*‑DyP4 decolorizing rate for dyes. In other words, three variants, W264F, W264H and W264Y, maintained good catalytic activity toward these substrates among eight variants of W264.

#### Steady-state kinetic constants of the W264 variants

Because the oxidative ability was lost in variants W264D, W264E, W264G, W264L, as shown in Fig. 6, the steady-state kinetic constants were measured among variants W264F, W264H, W264R and W264Y. According to the data in Table 3, the *k*_*cat*_/*K*_*m*_ of W264F and W264H for ABTS, DMP and RB 19 had a similar order of magnitude compared with the *k*_*cat*_/*K*_*m*_ of *Il*‑DyP4 (10^7^, 10^6^ and 10^6^, respectively), while the *k*_*cat*_/*K*_*m*_ of W264R and W264Y for ABTS and DMP was found to be 1–2 orders of magnitude lower than that of *Il*‑DyP4. The *k*_*cat*_/*K*_*m*_ of W264R and W264Y for RB 19 were not calculated because of very low enzyme activity. In particular, enzyme activity of W264Y decreased at higher concentrations of RB19, and an unusual steady-state kinetic behavior was observed. Overall, the kinetic parameters were consistent with the result from the oxidation and decolorization assays above.

**Table 3:** Steady-state kinetic constants (*K*_*m*_ (μM), *K*_*cat*_ (s ^−1^), *k*_*cat*_/*K*_*m*_ (M^−1^ s^−1^)) for the oxidation of ABTS, DMP and RB 19 by *Il*-DyP4 and the W264 variants

	Kinetic constants^a^	*Il*-DyP4^c^	W264F ^c^	W264H ^c^	W264R ^c^	W264Y ^c^
ABTS	*K*_*m*_	67.29 ± 13.05	71.87 ± 20.86	68.90 ± 14.06	19.47 ± 4.41	62.30 ± 14.99
	*k*_*cat*_	790 ± 56.06	384.85 ± 42.27	684.34 ± 52.02	52.78 ± 2.86	243.13 ± 23.59
	*k*_*cat/*_*K*_*m*_	(1.17 ± 0.43) × 10^7^	(5.35 ± 2.02) × 10^6^	(9.93 ± 3.70) × 10^6^	(2.71 ± 0.65) × 10^6^	(3.90 ± 1.57) × 10^6^
DMP	*K*_*m*_	8.98 ± 1.02	229.05 ± 30.78	42.64 ± 6.32	1082.00 ± 283.00	953.00 ± 102.70
	*k*_*cat*_	58.99 ± 0.45	71.21 ± 2.60	54.29 ± 1.08	18.28 ± 2.30	56.25 ± 2.81
	*k*_*cat/*_*K*_*m*_	(6.57 ± 0.44) × 10^6^	(3.11 ± 0.84) × 10^5^	(1.27 ± 0.17) × 10^6^	(1.69 ± 0.81) × 10^4^	(5.90 ± 2.74) × 10^4^
RB19	*K*_*m*_	139.87 ± 42.85	27.60 ± 7.70	66.50 ± 25.31	–^b^	–^b^
	*k*_*cat*_	187.90 ± 37.00	46.11 ± 5.91	86.87 ± 19.20	–^b^	–^b^
	*k*_*cat/*_*K*_*m*_	(1.34 ± 0.86) × 10^6^	(1.67 ± 0.76) × 10^6^	(1.31 ± 0.76) × 10^6^	–^b^	–^b^

^a^All data were fitted using the hyperbola function by Origin

^b^Not detected

^c^Substrate oxidation was measured at pH 3.5 (10 mM sodium tartrate buffer) using 3.3 nM of enzyme concentration obtained from a molar extinction coefficient

#### Spectral characterization of the W264 variants

**Fig. 7:**
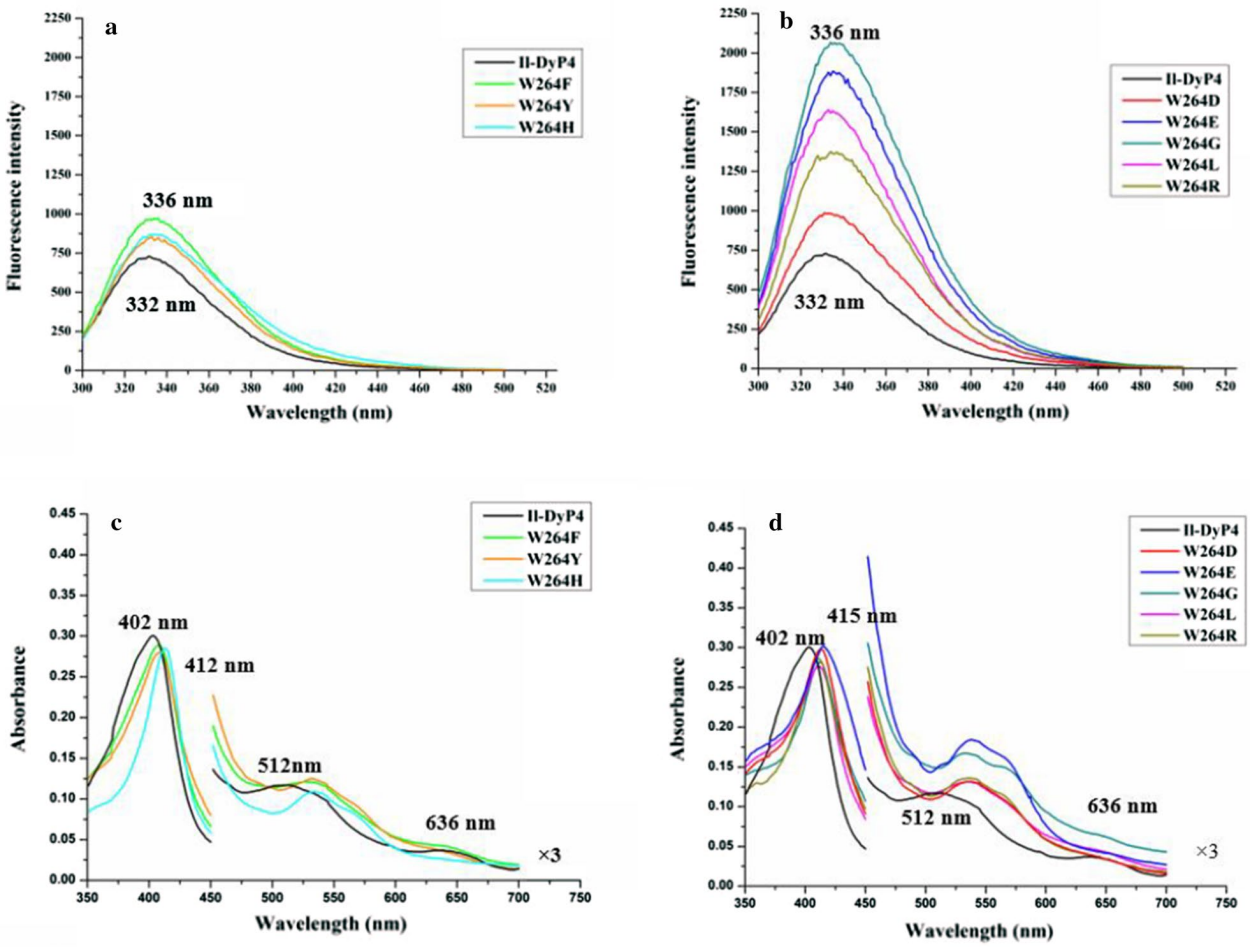
The spectral characterizations of *Il*-DyP4 and the W264 variants. **a**, **b** Fluorescence spectra. **c**, **d** UV–Vis absorbance spectra. All samples used in these assays had 0.1–0.11 mg/mL of each of enzymes in 10 mM sodium acetate buffer, pH 6.0

The UV–vis absorbance spectra and fluorescence spectra of each of the variants and *Il*‑DyP4 were measured, respectively, to investigate the role of W264 in *Il*‑DyP4 protein. As the fluorescence spectra in Fig. 7a, b show, all W264 variants showed a degree of redshift (about 4 nm) in maximum emission wavelengths (*λ*_max_) and an obvious increase in fluorescence intensity (*I*_max_), compared with that of *Il*‑DyP4. Meanwhile, the Soret band of all W264 variants also showed an obvious shift of about 10 nm, compared with that of *Il*‑DyP4 (Fig. 7c, d). The inactivated variants W264D, W264E, W264G and W264L showed the disappearance of characteristic charge transfer band at ~ 636 nm (CT1) that existed in *Il*‑DyP4 and the active variants (W264F, W264H and W264Y). The heme *b* contents of these variants were about 0.68–1.11 mol/mol of protein, similarly to the *Il*‑DyP4 (~ 1.01 mol/mol of protein). The above results indicated that the replacement of W264 by other residues, especially those residues without bulky side-chain, led to the change of heme microenvironment structure and spatial conformation of the protein.

#### EPR spectrum of the W264 variants at resting state

**Fig. 8:**
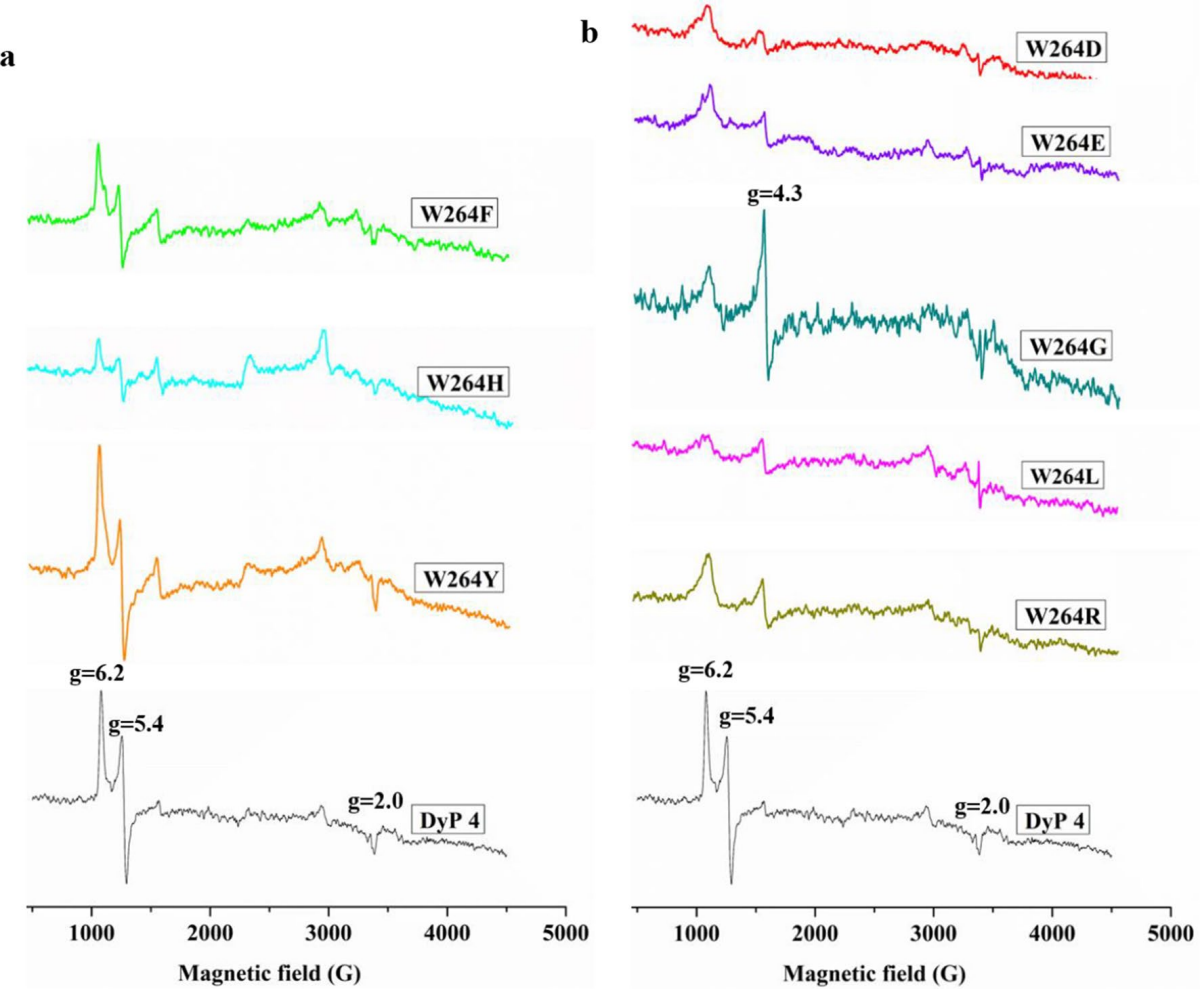
The EPR spectra of *Il*-DyP4 and the W264 variants. **a** W264F, W264H, W264Y and *Il*-DyP4. **b** W264D, W264E, W264G, W264L, W264R and *Il*-DyP4. The samples used in the assay contained 40 μM of the purified enzymes in sodium acetate buffer, pH 6.0

To further analyze the influence of W264 on the heme pocket microenvironment of *Il*‑DyP4, the EPR spectra of the W264 variants at resting state were obtained and compared with that of *Il*‑DyP4. As shown in Fig. 8, the active variants of W264F, W264H and W264Y (Fig. 8a) exhibited the signal of reactive high-spin Fe^3+^ (*g* = 6.2 and *g* = 5.4) in the magnetic field range of 1000–1500 G, the same as *Il*‑DyP4. Conversely, the same ferric species disappeared in the EPR spectra of the inactivated variants, namely, W264D, W264E, W264G, W264L and W264R (Fig. 8b). In addition, obvious signals of free Fe^3+^ appeared in the EPR spectrum of variant W264G (*g* = 4.3), as shown in Fig. 8b. Hence, it can be concluded that inactivation of the variants W264D, W264E, W264G, W264L and W264R mainly resulted from the disappearance of high-spin heme Fe^3+^. In other words, W264 plays an important role in maintaining the high-spin state of heme Fe^3+^ in *Il*‑DyP4.

### Investigation of the role of W380 in *Il*‑DyP4

#### Oxidative activity and decolorizing rate of the W380 variants

**Fig. 9:**
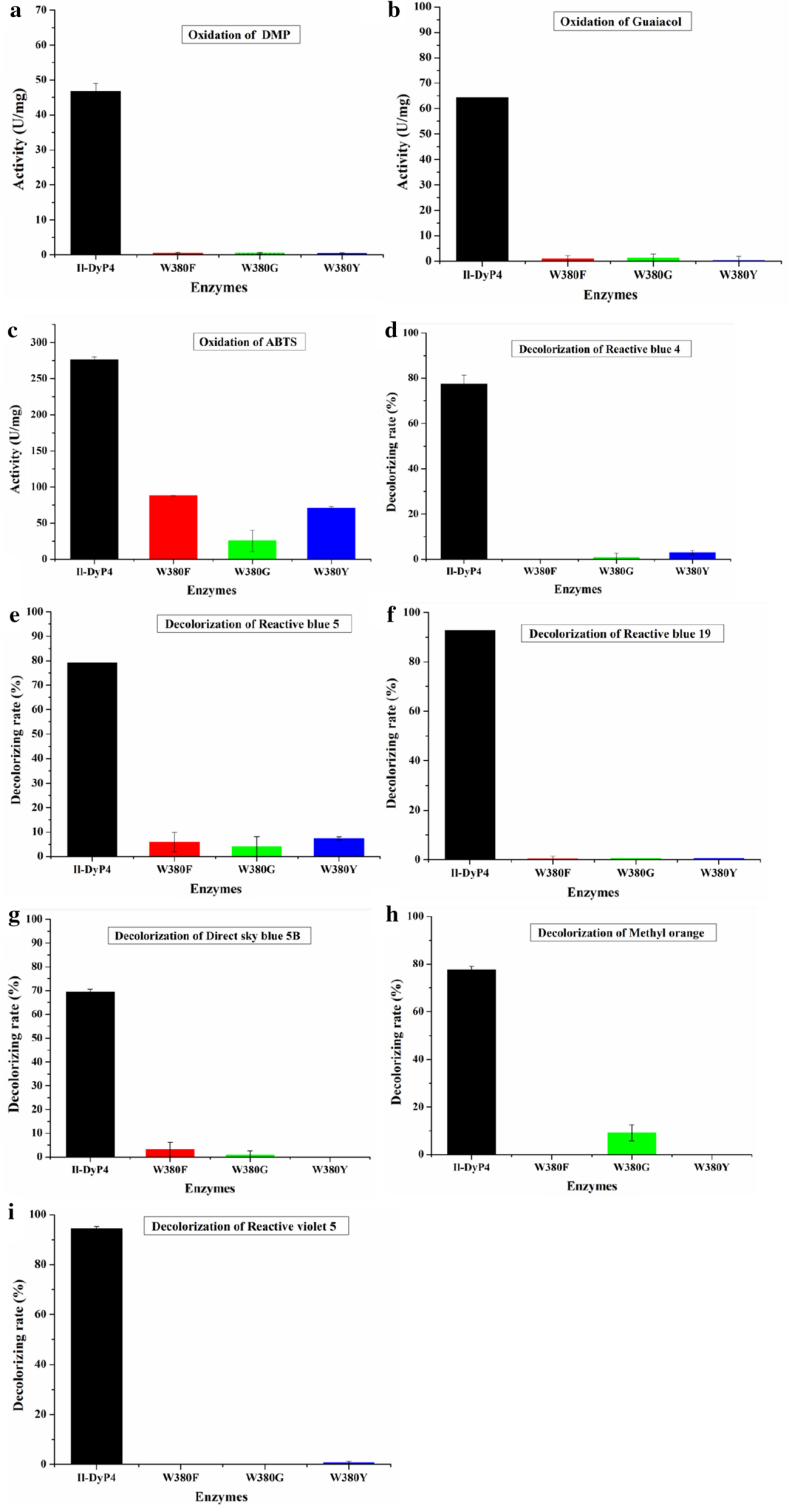
The oxidative activity and decolorizing rate of *Il*-DyP4 and the W380 variants. **a–c** The oxidative activity for DMP, guaiacol and ABTS, respectively. **d–i** The decolorizing rate for RB 4, RB 5, RB 19, 5B, MO and RV 5, respectively. The assay mixtures (1 mL) contained 1 mM of DMP, guaiacol and ABTS or 25–200 μM of dyes, 0.25 μg/mL (for oxidation assays) or 0.5 μg/mL (for decolorization assays) of purified enzyme. The reaction was initiated by 0.1 mM H_2_O_2_ at 35 °C, pH 3.5. Samples without enzyme served as the control

To explore the role of W380, three amino acid residues, namely, F, G and Y were selected for the site-directed mutagenesis, and the variants W380F, W380G and W380Y were obtained. Phenylalanine was chosen because it is a less oxidizable amino acid that cannot form a stable radical. Glycine was chosen because it is the smallest side-chain group amino acid with a subtle steric effect. Tyrosine was selected because it is a common redox-active amino acid, which can form stable radicals like tryptophan. The UV–vis spectra of these variants revealed the characteristic Soret band (Additional file 1: Fig. S2b), and the disappearance of charge transfer band at ~ 636 nm (CT1). The heme *b* contents of the variants W380F, W380G and W380Y were 0.87–0.98 mol/mol of protein and were close to 1.01 of *Il*‑DyP4. As shown in Fig. 9, all variants showed decreased oxidative activity and decolorizing ability (more than 95% of activity lost for most of the substrates) compared with that of *Il*‑DyP4. These results indicated that W380 performs an irreplaceable function in *Il*‑DyP4 catalysis.

#### EPR spectrum of the W380 variants at activated state

**Fig. 10:**
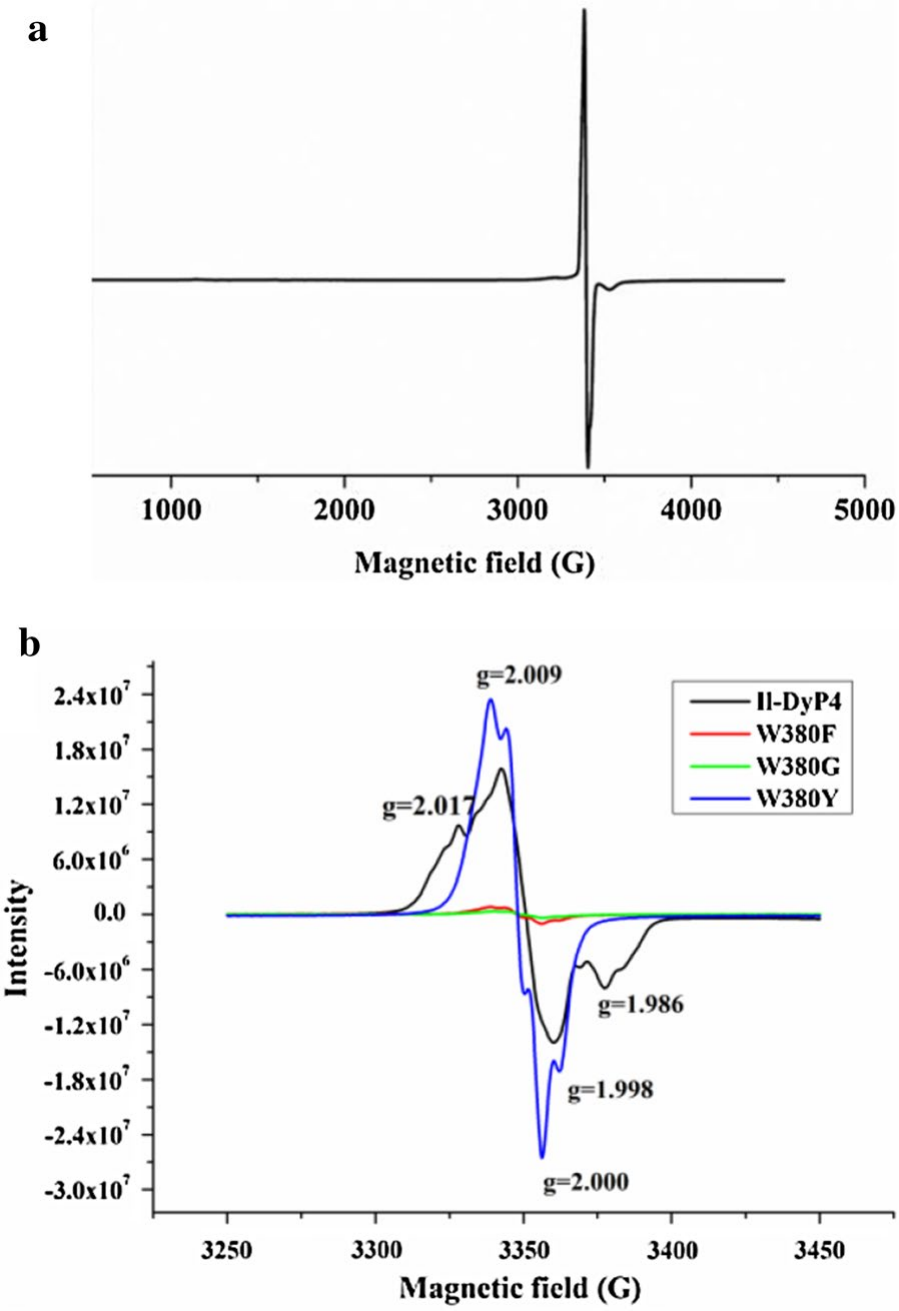
The EPR spectra of *Il*-DyP4 and the W380 variants. **a** Wide scan spectra of *Il*-DyP4 after the addition of H_2_O_2_ (10 eq). **b** Narrow scan spectra of *Il*-DyP4 and the W380 variants after the addition of H_2_O_2_ (10 eq). The samples used in the assay contained 40 μM of the purified enzymes in 10 mM sodium acetate buffer, pH 6.0

The EPR spectrum of *Il*‑DyP4 in Fig. 10a showed that, with the addition of H_2_O_2_, a protein-based radical signal with unresolved hyperfine couplings was generated (*g* = 2.0). To explore whether the W380 variants is able to generate the protein-based radicals like *Il*‑DyP4, the EPR spectra of each variant (40 μM) were recorded in the presence of 400 μM H_2_O_2_ at pH 6.0. Thus, a detailed picture of those radical signals by narrow scan EPR spectra is shown in Fig. 10b, it can be seen that the intensities of the radical signals of the variants W380F and W380G are much weaker than that of *Il*‑DyP4. Intriguingly, although the variant W380Y showed very low enzymatic activity in the above substrate oxidation and decolorization assays, it exhibited a radical signal with slightly enhanced strength compared with *Il*‑DyP4. However, in any case, the EPR spectra showed the key role of W380 in protein-centered radical generation, which is essential for the catalytic function of *Il*‑DyP4.

#### Steady-state kinetic constants of the W380 variants

For further analysis of the radical-forming variant W380Y, the steady-state kinetic constants of W380Y were measured and compared with the data from *Il*‑DyP4. The data listed in Table 4 show that the reduction of H_2_O_2_ (using H_2_O_2_ as electron acceptor) by W380Y exhibited a *k*_*cat*_/*K*_*m*_ value (8.25 × 10^6^) close to that of *Il*‑DyP4 (1.21 × 10^7^), although *K*_*m*_ of W380Y for H_2_O_2_ substrate decreased about 4 times. In comparison, the *k*_*cat*_/*K*_*m*_ of W380Y for ABTS oxidation decreased obviously by two orders of magnitude, and the catalytic activity of W380Y for DMP and RB 19 oxidation was lost. Therefore, the above data demonstrated that the mutation of W380 by tyrosine did not weaken the first step of the catalytic cycles (oxidation of the resting state Fe^3+^ by H_2_O_2_) significantly, but it weakened the oxidation of ABTS and other reducing substrates which has a larger size than H_2_O_2_.

**Table 4:** Steady-state kinetic constants (*K*_*m*_ (μM), *k*_*cat*_ (s ^−1^), *k*_*cat*_/*K*_*m*_ (M^−1^ s^−1^)) for the reduction of H_2_O_2_ and the oxidation of ABTS, DMP and RB 19 by *Il*-DyP4 and the variants W380Y

	Kinetic constants^a^	H_2_O_2_	ABTS	DMP	RB19
*Il*-DyP4^c^	*K*_*m*_	84.48 ± 20.57	67.29 ± 13.05	8.98 ± 1.02	139.87 ± 42.80
	*k*_*cat*_	1025.00 ± 37.94	790.00 ± 56.06	58.99 ± 0.45	187.9 ± 37
	*k*_*cat/*_*K*_*m*_	(1.21 ± 0.18) × 10^7^	(1.17 ± 0.43) × 10^7^	(6.57 ± 0.44) × 10^6^	(1.34 ± 0.86) × 10^6^
W380Y^c^	*K*_*m*_	20.08 ± 3.35	275.07 ± 77.79	–^b^	–^b^
	*k*_*cat*_	165.65 ± 8.28	88.38 ± 15.66	–^b^	–^b^
	*k*_*cat/*_*K*_*m*_	(8.25 ± 2.47) × 10^6^	(3.21 ± 2.01) × 10^5^	–^b^	–^b^

^a^All data were fitted using the hyperbola function by Origin

^b^Not calculated because of a lack of activity

^c^Substrate oxidation was measured at pH 3.5 (10 mM sodium tartrate buffer) using 3.3 nM of enzyme concentration obtained from a molar extinction coefficient

## Discussion

Nowadays, increasing attention has been paid to DyPs because these enzymes exhibit great potential for a variety of biochemical processes, such as the decolorization of industrial dyes, depolymerization of lignin, biological refining and bioremediation [33–38]. Therefore, it is necessary to study the enzyme structure and the key residues involved in *Il*‑DyP4 catalysis.

The crystal structure showed that *Il*‑DyP4 possessed the typical α + β protein structure of the DyPs family, the heme active center and the heme access channels. Compared with the two other reported fungal DyPs structures, *Bad*DyP from *B. adusta* and *Aau*DyP from *A. auricula-judae*, the *Il*‑DyP4 structure is similar to them with an equal number of α-helices (Additional file 1: Fig. S3a–c) and the same composition of residues (Asp, Arg, Leu and Phe) in the pocket structure for the conversion of H_2_O_2_ into H_2_O (Additional file 1: Fig. S3d–f). However, the number of β-strands is a little bit different compared with the other two: there are 7, 11 and 15 β-strands in *Il*‑DyP4, *Aau*DyP and *Bad*DyP, respectively. In addition, the first X in the conserved motif GXXDG is Tyr in *Il*‑DyP4 while it is Phe at the same position in *Aau*DyP and *Bad*DyP. In *Il*‑DyP4, two channels from the enzyme molecular surface to the heme region were found. The main channel (channel 1), as what has already been observed in *Bad*DyP, *Aau*DyP and bacterial *Kp*DyP structures [18, 27, 39], was suggested as the access channel of H_2_O_2_. However, the channel is too narrow to allow the entrance of various macromolecular substrates, especially dyes. We found another channel, channel 2 (from the surface leads to the heme propionate), in *Il*‑DyP4. The channel 2 is somewhat analogous to the Mn^2+^ entrance channel in VPs and MnPs [34], but it is different from the reported Mn^2+^ oxidation site in *Pleurotus ostreatus* DyP4 (*Pos*DyP4) that is located at the surface of the protein [40].

The chemical modification of tryptophan residues by NBS had confirmed that tryptophan residues are associated with most of the *Il*‑DyP4 enzymatic activity. Subsequent oxidative and decolorizing characterization of the variants (replacing Trp residues with Phe, Fig. 5) indicated that W264 and W380 play more important roles in *Il*‑DyP4. Although the enzymatic activity of W264F did not decrease as sharply as what has been observed in W380F, W264 has a short distance from heme cofactor (Additional file 1: Fig. S4a) among the five tryptophan residues, and its location in *Il*‑DyP4 is equivalent to the W264 in *Bad*DyP and W256 in *Aau*DyP (Additional file 1: Fig. S4b–d). Moreover, W264 is conserved among five DyPs from *I. lacteus* F17 [32]. Therefore, W264 was selected together with W380 for the mutagenesis with a replacement by different amino acids in the subsequent studies to further explore their respective functions in *Il*‑DyP4.

To study the role of W264 in *Il*‑DyP4, we replaced the residue with D, E, F, G, H, L, R and Y. The results from oxidation assays, decolorization assays (Fig. 6) and steady-state kinetic constants (Table 3) showed that only variants W264F, W264H and W264Y still possess catalytic ability toward various substrates, while the catalytic ability of variants W264D, W264E, W264G, W264L and W264R was almost lost. Fluorescence spectra showed the difference in protein conformation between the *Il*‑DyP4 and the variants of W264. Compared with *Il*‑DyP4, the redshift of about 4 nm in λ_max_ of the W264 variants (Fig. 7a, b) suggested that the tryptophan residues in the protein were more exposed to a hydrophilic environment, indicating the substitution of W264 changed the spatial structure of protein [41–43]. In addition, compared with that of *Il*‑DyP4, UV–vis absorbance spectra (Fig. 7c, d) of the inactivated variants W264D, W264E, W264G and W264L showed the lack of the characteristic charge transfer bands at ~ 637 nm (CT1), indicating that a heme pocket microenvironment of these variants were changed [42, 44]. More importantly, the results of EPR experiments showed that the inactivated variants (W264D, W264E, W264G, W264L and W264R) lost their high-spin Fe^3+^, while the active variants (W264F, W264H and W264Y) exhibited the same signal of reactive high-spin Fe^3+^ as *Il*‑DyP4. By further analysis of the structural characteristics of the amino acids involved in the mutagenesis of W264, we found that those amino acids (F, H and Y) used in mutagenesis to produce redox-active variants all have a bulky side-chain group with stronger steric hindrance like W264 (indole ring, Additional file 1: Fig. S5), implying that the bulky side-chain group of W264 is of great significance to the catalytic ability of *Il*‑DyP4 for the maintenance of the normal protein conformation and the high-spin state of Fe^3+^. Indeed, from Fig. 8b, W264G displayed the obvious signals of free Fe^3+^ in the EPR spectrum due to the side-chain group of G is too small to provide enough steric hindrance for maintaining the natural environment of heme Fe^3+^. The presence of enzyme activity of the variants W264F, W264H and W264Y could be attributed to the bulky side-chain groups of F, H and Y.

As we know, in DyPs, the proximal conserved histidine is important for constraining the Fe^3+^ in the heme plane and stabilizing the Fe(IV) intermediate [6]. The Fe^3+^ heme is coordinated to the protein through the imidazole group of this axial histidine. However, the crystal structure of *Il*‑DyP4 showed that W264 is far away from the proximal conserved histidine residue (H312) (~ 10.4 Å, Additional file 1: Fig. S6a). Further analyses revealed that another critical residue phenylalanine (F261) is closely located in the proximal heme cavity, as shown in Additional file 1: Fig. S4b and S6b. It is interesting to note that the side-chain of F261 is stacked almost parallel to the proximal H312 (~ 4.9 Å, Additional file 1: Fig. S6b), indicating that the F261 is also critical for the stabilization of the heme pocket. Thus, we can see that W264 may interact with the proximal F261 to form a π-π stacking interaction, and with a nearby peptide bone to form a hydrogen bonding interaction. In addition, W264 is noted to locate on a loop between α-helices 14 and 15, according to the crystal structure of *Il*‑DyP4 (Fig. 1). Mutations at W264 may change the conformation of the loop, resulting in the movement of the two α-helices each other. Therefore, we demonstrated that W264 plays a role in stabilizing the heme pocket microenvironment and protein conformation.

In the investigation of the role of W380, the apparent loss in oxidative and decolorizing ability among all of the three W380 variants (Fig. 9) demonstrated that W380 performs an irreplaceable role in *Il*‑DyP4 catalysis. Moreover, the loss of enzyme activity was observed not only for bulky dye substrates but also for simple compounds like DMP, guaiacol and ABTS. The result is different from a *Pseudomonas putida* DyP that was suggested to oxidize ABTS and DMP at the heme access channel [22]. According to the surface-exposed location of W380 (Additional file 1: Fig. S7) and its same location with the identified exposed catalytic residue W373 in *Bad*DyP from *B. adusta* and W377 in *Aau*DyP from *A. auricula-judae* (Additional file 1: Fig. S3d–f), we hypothesize that W380 functions as the surface oxidation site with radical generation in *Il*‑DyP4. Thus, the EPR analysis was performed. The appearance of a radical-like signal for W380 and W380Y was evident, with the disappearance of the iron signal, while the reacting signal of the W380F and W380G, respectively, with H_2_O_2_, after 10 s was essentially EPR silent (Fig. 10b). Namely, the replacement of W380 with F and G, respectively, lost almost all of their radical-forming abilities. The negligible radical signals of the variants W380F and W380G might corresponded to the oxoferryl-porphyrin radicals of enzymes, similar to what was reported by Shrestha et al. and Ruiz-Dueñas et al. [45, 46]. Besides, it is worth noting that, for the variant W380Y, although tyrosine is a redox-active amino acid that can form radicals under oxidative conditions, the results showed that the substitution of W380 by residue Y caused enzyme inactivation. The steady-state kinetic constants (Table 4) showed that W380Y had decreased *K*_*m*_ for the reduction of H_2_O_2_ substrate compared with that of *Il*‑DyP4, while the oxidative ability for other substrates (ABTS, DMP and RB 19) decreased sharply. By comparing different side-chain groups between W (indolic ring) and Y (hydroxyphenyl), we consider that the decrease in hydrophobicity of residue Y could be one of the reasons for the enzyme inactivation. As shown in Additional file 1: Fig. S8, W380 is located in an extremely hydrophobic environment with five hydrophobic phenylalanines surrounding it, which might facilitate surface interactions between W380 and the substrates that are rich in aromatic moieties, such as dyes [46, 47]. Hence, the substitution of W380 by residue Y might disturb the hydrophobic environment of the surface oxidation site, which weakened the interaction between W380 and substrates. In addition, the relatively small solvent-accessible areas of residue Y, and the relatively weaker oxidative ability of tyrosyl radicals, compared with residue W, decreased the enzyme activity, as in the study by Shrestha et al. [45]. Shrestha et al. demonstrated that the catalytic function of an exposed tyrosyl radical in A-class *Tc*DyP is minimal compared with the catalytic residue W263 [45]. Interestingly, an EPR study of *Pleurotus eryngii* versatile peroxidase (VP) by Ruiz-Dueñas et al. [46], found that a tyrosyl radical yield was lower than the native enzyme VPI of tryptophanyl radical, revealing that the Trp-164 radical was involved in catalysis by VP. On the other hand, as observed in yeast cytochrome c peroxidase and lactoperoxidase, the formation of tyrosyl radicals can result in enzyme inactivation through intermolecular cross-linking [48, 49]. In short, this study analyzed the function of W380 of *Il*‑DyP4 and identified it as the surface-exposed residue that can generate redox-active radicals like other reported tryptophan residues in *Aau*DyP, LiP and VP [27, 50, 51]. Moreover, the loss of catalytic ability for all tested substrates after mutation demonstrated that W380 was responsible for the oxidation of not only bulky dyes but also simple compounds, which is different from those classical heme peroxidases that oxidize small compounds at either the δ or λ edges of the heme group [1, 34].

*Il*‑DyP4 possesses five Trp residues, which are shown in Additional file 1: Fig. S4a. Furthermore, we note that distance of residue to the heme iron varies, in the order of W147 (29 Å), W211 (28 Å), W109 (17 Å), W264 (12 Å), W380 (11 Å). In view of the fastest electron transport in *Il*‑DyP4 from radical-forming exposed residues to the H_2_O_2_-activated heme cofactor, W380 is the most likely candidate to serve as the origin of the organic radical; as a result, Cpd I could be rapidly reduced by obtaining electrons from tryptophanyl radical of W380. Interestingly, a case like this has been reported by Linde et al., a homologous surface site of Trp 377 in *Aau*DyP, with an electron-transfer pathway to the heme iron was identified by using quantum mechanics/molecular mechanics calculations [27]. From a structural superpositions of *Il*‑DyP4 (yellow, PDB: 7D8M) and *Aau*DyP (azure, PDB: 4W7J) (Additional file 1: Fig. S9), we consider that a similar electron transport pathway may be present in *Il*‑DyP4. It would initiate in *Il*‑DyP4 W380 and proceeds via P318, N317 and R314, to distal H312.

However, further verification of the radical contributions of W380 to the catalytic activity of *Il*‑DyP4, is now required by quantitative measurements of spin density of W380 using multi-frequency EPR spectra of H_2_O_2_-activated *Il*‑DyP4.

## Conclusion

In conclusion, we have solved the first validated crystal structure of *Il*‑DyP4 with one ferric heme molecule from *I. lacteus* F17, which was consistent with the structural features of the DyPs family, comprising the GXXDG motif, four amino acid residues for the H_2_O_2_ reaction, and several conserved residues (Asp172, Arg335, His312) located at the heme pocket region. In addition, W264 and W380 were identified as two important tryptophans with different functions in *Il*‑DyP4 through step-by-step in-depth studies: 1) W264 plays an important role in maintaining the normal spatial conformation and the high-spin state of heme Fe^3+^, to ensure the oxidative function of *Il*‑DyP4; 2) W380 was identified as the site of surface-exposed protein radical that is responsible for the oxidation of both bulky and simple compounds. In brief, the structure of *Il*‑DyP4 and the functions of tryptophan residues were studied thoroughly. However, the functions of other redox-active residue tyrosines with a high number (10 in total) in *Il*‑DyP4 remain unclear. Considering that classic ligninolytic peroxidases (LiPs, VPs and MnPs) are completely depleted of tyrosine residues in their sequences, the existence of rich tyrosine residues in *Il*‑DyP4 should have some specific significance. Hence, research on the role of tyrosine residues is in progress for further understanding of the catalytic mechanism of class V DyPs from fungi.

## Supplementary Information

**Additional file 1: Table S1** The primers sequences used in this study. **Table S2** The structures of substrates used in this study. **Fig. S1** Sequence alignments of tryptophan and tyrosine residues in *Il*-DyP4 with other representative class V DyPs. Highlighted residues include the following: (i) tryptophan residues were shown in *red* and the corresponding tryptophans in *Il*-DyP4 were indicated in the black box. (ii) tyrosine residues were shown in *green* and the corresponding tyrosines in *Il*-DyP4 were indicated in the black box. Above amino acid sequences of class V DyPs were downloaded from NCBI database: DyP2 from *Amycolatopsis* sp. (GI: 496374264), AnaPX from *Anabaena* sp. (GI: 1772287408), *Fm*DyP from *Fomitiporia mediterranea* (GI: 595785908), *Vv*DyP from *Volvariella volvacea* (GenBank: AKU04643.1), *Tv*DyP1 from *Trametes versicolor* (GI: 636616485), *Sh*DyP from *Stereum hirsutum* (GI: 597911761), *Hi*DyP from *Heterobasidion irregulare* (GI: 575067022), *Gl*DyP from *Ganoderma lucidum* (GenBank: ADN05763.1), *Ds*DyP from *Dichomitus squalens* (GI: 1585543766), *Aau*DyP from *Auricularia auricula-judae* (GI: 1048348430) and *Bad*DyP from *Bjerkandera adusta* (GI: 116666995). **Fig. S2** Electronic absorbance spectra of *Il*-DyP4 and the variants. **a** Absorbance spectra of *Il*-DyP4 and the W variants. **b** Absorbance spectra of *Il*-DyP4 and the W380 variants. All samples used in these assays had about 0.1–0.11 mg/mL of each of enzymes in 10 mM sodium acetate buffer, pH 6.0. **Fig. S3** The structure comparison between *Il*-DyP4 and other fungal DyPs. **a-c** represent *Il*-DyP4 (PDB: 7D8M), *Bad*DyP from *Bjerkandera adusta* (PDB: 3AFV) and *Aau*DyP from *Auricularia auricula-judae* (PDB: 4W7J), respectively. **d–f** show the important residues surrounding the heme of each protein and the suggested surface-exposed catalytic tryptophan is showed in orange. **Fig. S4** The location of W264 in *Il*-DyP4 and the comparison with other fungal DyPs. a represents the distances (Å) between the above tryptophan residues and the heme iron in *Il*-DyP4; b-d represent *Il*-DyP4, *Bad*DyP and *Aau*DyP, respectively, showing the position of W264 in *Il*-DyP4 and the corresponding residues in *Bad*DyP and *Aau*DyP. **Fig. S5** The structure comparison between W and those amino acids used in W264 mutation. **Fig. S6** a shows the distance between W264 and H312. b shows the F261 between W264 and H312. Yellow sticks represent the heme cofactor. **Fig. S7** Location of W380 in *Il*‑DyP4. The molecular surface is shown in white. The red sphere represents W380. **Fig. S8** The hydrophobic phenylalanines surrounding W380 in *Il*‑DyP4. The yellow sticks represent the heme cofactor. **Fig. S9** Structural superpositions of *Il*‑DyP4 (yellow, PDB: 7D8M) and *Aau*DyP (azure, PDB: 4W7J) showing a similar electron transfer pathway from the surface-exposed W380 to heme (according to Linde et al. 2015 [27]).

## Data Availability

All data generated or analyzed during this study are included in this published article and its additional files.

## Abbreviations

DyPs: Dye-decolorizing peroxidases
EPR: Electron paramagnetic resonance
LiPs: Lignin peroxidases
MnPs: Manganese peroxidases
VPs: Versatile peroxidases
H_2_O_2_: Hydrogen peroxide
Cpd I: Compound I
Por^+^•: Porphyrin cationic radical
Cpd II: Compound II
EDTA: Ethylene diamine tetraacetic acid
SDS-PAGE: Sodium dodecyl sulfate polyacrylamide gel electrophoresis
NBS: *N*-Bromosuccinimide
DMP: 2,6-Dimethoxyphenol
ABTS: 2,2’-Azino-bis(3-ethylbenzthiazoline-6-sulfonic acid)
RB 4: Reactive blue 4
RB 5: Reactive blue 5
RB 19: Reactive blue 19
5B: Direct sky blue 5B
MO: Methyl orange
RV 5: Reactive violet 5
W: Tryptophan
D: Aspartic acid
E: Glutamic acid
F: Phenylalanine
G: Glycine
H: Histidine
L: Leucine
R: Arginine
Y: Tyrosine
