# Supplementary material for: Revealing two important tryptophan residues with completely different roles in a dye-decolorizing peroxidase from Irpex lacteus F17
Source: Biotechnol Biofuels. 2021 May 31;14:128. doi: 10.1186/s13068-021-01978-y (PMC8165797; doi:10.1186/s13068-021-01978-y)
Supplement: Supplementary file 1 — Additional file 1: Table S1 The primers sequences used in this study. Table S2 The structures of substrates used in this study. Fig. S1 Sequence alignments of tryptophan and tyrosine residues in Il-DyP4 with other representative class V DyPs. Highlighted residues include the following: (i) tryptophan residues were shown in red and the corresponding tryptophans in Il-DyP4 were indicated in the black box. (ii) tyrosine residues were shown in green and the corresponding tyrosines in Il-DyP4 were indicated in the black box. Above amino acid sequences of class V DyPs were downloaded from NCBI database: DyP2 from Amycolatopsis sp. (GI: 496374264), AnaPX from Anabaena sp. (GI: 1772287408), FmDyP from Fomitiporia mediterranea (GI: 595785908), VvDyP from Volvariella volvacea (GenBank: AKU04643.1), TvDyP1 from Trametes versicolor (GI: 636616485), ShDyP from Stereum hirsutum (GI: 597911761), HiDyP from Heterobasidion irregulare (GI: 575067022), GlDyP from Ganoderma lucidum (GenBank: ADN05763.1), DsDyP from Dichomitus squalens (GI: 1585543766), AauDyP from Auricularia auricula-judae (GI: 1048348430) and BadDyP from Bjerkandera adusta (GI: 116666995). Fig. S2 Electronic absorbance spectra of Il-DyP4 and the variants. a Absorbance spectra of Il-DyP4 and the W variants. b Absorbance spectra of Il-DyP4 and the W380 variants. All samples used in these assays had about 0.1–0.11 mg/mL of each of enzymes in 10 mM sodium acetate buffer, pH 6.0. Fig. S3 The structure comparison between Il-DyP4 and other fungal DyPs. a-c represent Il-DyP4 (PDB: 7D8M), BadDyP from Bjerkandera adusta (PDB: 3AFV) and AauDyP from Auricularia auricula-judae (PDB: 4W7J), respectively. d–f show the important residues surrounding the heme of each protein and the suggested surface-exposed catalytic tryptophan is showed in orange. Fig. S4 The location of W264 in Il-DyP4 and the comparison with other fungal DyPs. a represents the distances (Å) between the above tryptophan residues and the heme iron in Il-DyP4; b-d r [file 13068_2021_1978_MOESM1_ESM.pdf]

Table S1 The primers sequences used in this study

Primers	Sequences
W109F	GGT GAT GAT ACC AGT ACC **TTC**[a] GAA GAA GCA T
	A TGC TTC TTC **GAA** GGT ACT GGT ATC ATC ACC
W147F	GGT GAT GCA **TTC** ACC ATT GTG TAT GA
	C ATA CAC AAT GGT **GAA** TGC ATC ACC A
W212F	GTT ATG AAT CGT CCG AGT **TTC** GCA CTG GA
	GCC ATC CAG TGC **GAA** ACT CGG ACG A
W264F	AGC CGC ATG TTT GGT CGT **TTC** AAA AGC
	GG TGC GCC GCT TTT **GAA** ACG ACC AA
W264D	AGC CGC ATG TTT GGT CGT **GAT** AAA AGC
	GG TGC GCC GCT TTT **ATC** ACG ACC AA
W264G	AGC CGC ATG TTT GGT CGT **GGC** AAA AGC
	GG TGC GCC GCT TTT **GCC** ACG ACC AA
W264Y	AGC CGC ATG TTT GGT CGT **TAT** AAA AGC
	GG TGC GCC GCT TTT **ATA** ACG ACC AA
W264H	AGC CGC ATG TTT GGT CGT **CAC** AAA AGC
	GG TGC GCC GCT TTT **GTG** ACG ACC AA
W264R	AGC CGC ATG TTT GGT CGT **CGT** AAA AGC
	GG TGC GCC GCT TTT **ACG** ACG ACC AA
W264E	AGC CGC ATG TTT GGT CGT **GAG** AAA AGC
	GG TGC GCC GCT TTT **CTC** ACG ACC AA

W264L	AGC CGC ATG TTT GGT CGT **CTC** AAA AGC
	GG TGC GCC GCT TTT **GTG** ACG ACC AA
W380F	CGT TTT CAG CAG CAG GCA **TTC** GCC AAT AA
	CG CGG ATT ATT GGC **GAA** TGC CTG CTG CT
W380D	CGT TTT CAG CAG CAG GCA **GAT** GCC AAT AA
	CG CGG ATT ATT GGC **ATC** TGC CTG CTG CT
W380G	CGT TTT CAG CAG CAG GCA **GGC** GCC AAT AA
	CG CGG ATT ATT GGC **GCC** TGC CTG CTG CT
W380Y	CGT TTT CAG CAG CAG GCA **TAT** GCC AAT AA
	CG CGG ATT ATT GGC **ATA** TGC CTG CTG CT

[a] The bold sequences indicate the new sequences after site-directed mutagenesis.

Table S2 The structures of substrates used in this study

Substrates	Type	Mr	Structure
2,6-dimethoxyphenol	Phenol	154.16	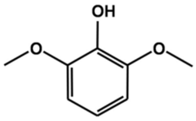
Guaiacol	Phenol	124.14	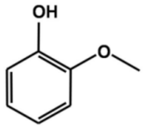
2,2’-azino-bis(3-ethylbenzthiazoline-6-sulfonic acid)	Aromatics	548.68	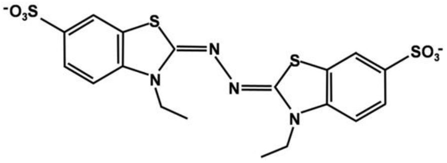
Reactive blue 4	Anthraquinone	637.43	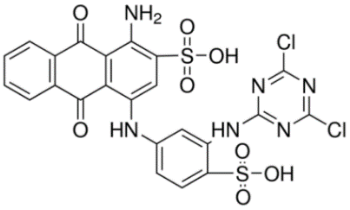
Reactive blue 5	Anthraquinone	774.17	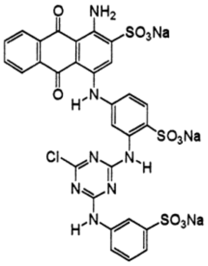
Reactive blue 19	Anthraquinone	626.54	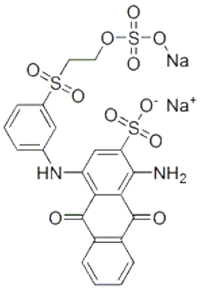
Direct sky blue 5B	Azo	992.8	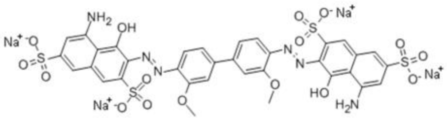
Methyl orange	Azo	327.33	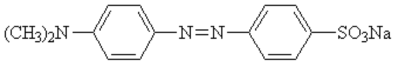
Reactive violet 5	Azo	735.58	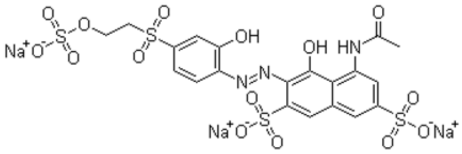

```
DyP2        ------------------------------------------------------------   0
AnaPX       --------------------MALTEKDLKHLPED-------------GIDSENPGKYRNL   27
AauDyP      ---------------------------------------------------------SLN   3
Il-DyP4     MQLRHFLAATAAFSAISQSSLAYHVKRARSTPLIGSFPGQPPLPTIAQVQSTSAGNDSLP   60
BadDyP      -----------------------------------------------------ANDTILP   7
FmDyP       ----------------------------------------------------MSWPTPAD   8
VvDyP       --------------------------------------MSQATQTAASNIPAPDTGDRLD   22
Pleos-DyP   --------------------------------------------------------PPLD   4
TvDyP1      -----------------------------------------------------MSSDALN   7
ShDyP       --------------------------------------------------MSSTTDQPLD   10
HiDyP       --------------------------------------------------MTTHTPAPLN   10
GlDyP       ---------------------------------------------------MASTLPPFN   9
DsDyP       ---------------------------------------------------MSATLTPLN   9

                                                   Y45
DyP2        --MQAGDLEPPEPQ----------------------------------------------   12
AnaPX       LNDLQGNILKGHGRDHSVHLFLQFKPEQVEVVKQWIQNFAQTYITSAKKQSDEAFK----   83
AauDyP      TDDIQGDILVGMHKQKQLFYFFAIND--PATFKTHLASDIAPVVAS-VTQ----------   50
Il-DyP4     FENIQGDILVGMKKDKEKFVFFHINN--ATAFKSVLKTYAPANITSVATI----------   108
BadDyP      LNNIQGDILVGMKKQKERFVFFQVND--ATSFKTALKTYVPERITSAAIL----------   55
FmDyP       LDDLQGDVVLGLPKRAETFIFFNIAN--VANFKKSLQQFIPAVTTTTQVQQLRKEIADHK   66
VvDyP       LTNIQGDILSGLPKKTETYYFFEITD--PATFKTHMKRFIREIKTVKGVLKDREAIERHR   80
Pleos-DyP   LNNIQGDILGGLPKRTETYFFFDVTN--VDQFKANMAHFIPHIKTSAGIIKDREAIKEHK   62
TvDyP1      FDDIQGDILVGLPKRVQQYIIFQIGSN-VAGFKQALTQLLPLITTTTQAMQNRAAIAANK   66
ShDyP       FDNIQGEILPGFPKPSLTYVLFQITN--AKAFRTALADLVPLITSTAQVMGHRQTIAHHK   68
HiDyP       LANVQGDILAGLPKKTQTYVLFEITD--AQAFRTALTHLIPLITSTEQVKKDRHSIDENK   68
GlDyP       PANVQGDILVGLPKKVQHYLFFQIDDD-VTAFRKRLHLLIPLITTTAQVQDDRAKIAANK   68
DsDyP       PANVQGDILVGLPKKVQHYLFFQIDND-VTSFRKRLGLLIPLITTTAQVQDDRAKISANK   68
                 *::     :

DyP2        -AVSAPLTRAAIFLMVQVNAGAEQI-------AK-DLLSDLA----GLQRA--VGFRDAG   57
AnaPX       ------YRQKGIPGQVFGNFFLSRHGYEYLEIEPFQIPGDKPFRMGMKNEEIRTSLGDP-   136
AauDyP      ------LSNVATQPLVALNIAFSNTGLLALGVTD-N-LGDSLFANGQAKDA--TSFKES-   99
Il-DyP4     ------IGPVANQPLAFVNLAFSHAGFGALNVTD-D-LQDTAFSDGQFKDS--PNLGDD-   157
BadDyP      ------ISDPSQQPLAFVNLGFSNTGLQALGITD-D-LGDAQFPDGQFADA--ANLGDD-   104
FmDyP       L-----TGSPDLLQIQCMNFALSRRGMKELGVTD-D-LRDGPFNDGQKAHA--QPLGDNG   117
VvDyP       KEHSKDGRKPPLIPLVGVNISFSHFGLAALEIDDGN-LVDTAFLSGQRADA--ENLGDAG   137
Pleos-DyP   R-----QKKPGLVPMAAVNVSFSHLGLQKLGITD-D-LSDNAFTTGQRKDA--EILGDPG   113
TvDyP1      KAAQEQGKTPELLKMSGVNIAFSHVGLAALGIND-N-IHDDLFTNGQQADA--QSLGDPG   122
ShDyP       AKVAQNKSEPGMLDIVCVNVAFSQTGLTTVHIK------DPSFVNGQLSDA--NDLGDTG   120
HiDyP       KTAAHSGHPPPLLDIVGVNVSFSQLGLNKLGIKD-D-IKDAVFKAGQLSDA--QNLGDPG   124
GlDyP       KKAAELGKAPELLRLSGVNIAFSQFGLTKLGIKD-D-MGDTAFKSGQLNDT--PNLGDAG   124
DsDyP       EAAAKEGKAPELLKLSGVNIAFSQFGLTKLGITD-N-VGDTAFVEGQLKDS--QNLGDAG   124
                              *   ..               *                : :
```

```
                            W109                         Y135             W147
DyP2           AGLTCVAGISSAAWDRIYGGPRPAELHELPVFAGEKHTSVLTAEADLLFHLRAERLDLCF      117
AnaPX          ---------KIETWDIGFQN----EIHALILLADDDIIDLLQIVNQMTQELRL------I      177
AauDyP         ----------TSSWVPQFAG---TGIHGVIILASDTTDLIDQQVASIESTFG-----SSI      141
Il-DyP4        ----------TSTWEEAFKG---TNVDGVFLIGSNDESITAQYRDDLNAKFG-----DAW      199
BadDyP         ----------LSQWVAPFTG---TTIHGVFLIGSDQDDFLDQFTDDISSTFG-----SSI      146
FmDyP          KQS---LLSFDPDWLLPFKN----EVDGVFLITGDSDSTVLDGINKVNNTF-----SGSF      165
VvDyP          TGT---GQDFVPDWEEPFRD---LHIHGVILIAGDSHGTVIKKLREIEALFDVKGSSPSI      191
Pleos-DyP      SKN---GDAFTPAWEAPFLK----DIHGVIFVAGDCHGSVNKKLDEIKHIFGVGTSHASI      166
TvDyP1         TSSGFPISHFTPSWDPAFLN----KTHGVIIIAGDSDDTVASVRKQVEAIFNVGGFNATL      178
ShDyP          SFES--GTFVPSDWLDAFRN----PVHGAIIISGPTPIVIRNKVNDVENMFGVHSGHSSL      174
HiDyP          TTNA--SKKFVPDWIDAFKK----GVHGVFIVSGSCHELVSKKVEEIEHIFGVHGHHASY      178
GlDyP          TTV---NNKFVPNWINAFKN----QIHGVVIISGDCDLTVAATQATVLGIFNI-GVRITL      176
DsDyP          TTDA--QGNFTPDWLPAFKN----QIHGLIIISGDSELTVSATQATVMAIFNI-GIHITL      177
                            *   :        .   .. .            :   :

                      Y151                Y170
DyP2           ELESLIMDRLRGSVQVLDEVQGFRYFDARDVLGFVDGTENPTGNGVNQA-----------      166
AnaPX          A----EIVHREDGFIL-----RNQSGQIIEHFGFVDGVSQPLFMKRDV--------VKER      220
AauDyP         S----KLYSLSA--SI-----RPGNEAGHEMFGFLDGIAQPAINGFNT-PLPGQNIVDAG      189
Il-DyP4        T----IVYDLDS--AA-----RPGNEKGHEHFGYLDGISNPTIPGFGT-PHPGQAVVDPG      247
BadDyP         T----QVQALSG--SA-----RPGDQAGHEHFGFLDGISQPSVTGWETTVFPGQAVVPPG      195
FmDyP          T----EMLKVEG--RV-----RPGKEKGHEHFGFRDGISFPAIRGFV-DPFPGQTQVDPG      213
VvDyP          K----EVTTIVG--DV-----RPGDVSAHEHFGFLDGISNPAVIGFDTRFHPGPVPVRPG      240
Pleos-DyP      S----EVTHVRG--DV-----RPGDVHAHEHFGWLDGISNPAVEQFDQNPLPGQDPIRPG      215
TvDyP1         S----EVITLSG--SV-----RPGDQKGHEHFGFMDGISQPAVQGVDTSPNPGQDTVHQG      227
ShDyP          K----VVLELVG--QP-----RPKPHNGHEHFGFLDGISHPAVEGFDITPNPGQQTVPQG      223
HiDyP          T----QLLSIVG--DV-----RPGKEKGHEHFGFLDGISQPAVKGIDTDPNPGQETIRQG      227
GlDyP          H----EVLTLKG--VV-----RPGDQKGHEHFGFLDGISQPAVKDFDTKPNPGQETVRQG      225
DsDyP          H----EVTTLKG--VV-----RPGAEKGHEHFGFLDGISQPAVKEFDTKPNPGQETVRQG      226
                     :                      : :*: **   *

                                          W212                 Y234
DyP2           VLV------------------DDDEPFNR------GSYVVVQKYLHDMAAWNALT-----      197
AnaPX          VNNCDFDKWDPKAPLDSILVEDP----NGNTKDSYGSYLVYRKLEQNVKAFREDQRKLAQ      276
AauDyP         VIITGATN-------DP-I--------TRPSWAVGGSFLAFRQLEQLVPEFNKYLLDNAP      233
Il-DyP4        IIFTGRSK-------DPVM--------NRPSWALDGSFLVFRKLKQLVPEFNKYVLDNAL      292
BadDyP         IILTGRDG-------DT-G--------TRPSWALDGSFMAFRHFQQKVPEFNAYTLANAI      239
FmDyP          VIVCKTNG-------DGVL--------FRPDWAKNGSFLVYRHLQQFVPEFNTFVENNPV      258
VvDyP          AILVGRDG-------DSNEP-------NRDSWMIDGSFMVFRYLFQKVPEFDKFLEDNAI      286
Pleos-DyP      FILAKENG-------DSRAA-------ARPDWAKDGSFLTFRYLFQMVPEFDDFLESNPI      261
TvDyP1         VILCKRDN-------DNTS-------LLRPAWAKDGSFLVLRYLFQLVPEFNVFLQSNPI      273
ShDyP          TILLGRLG-------DEI---------IRPKWAVDGSFLALRHLSQLVPEFDEFLIDNPI      267
HiDyP          IMLLGRDG-------DDVAA-------QRPSWALDGSFLAFRYLFQLVPEFNKFLHDNPI      273
GlDyP          VILCGREG-------DVVAGSKPEQPFVRPAWALDGSFLALRYLFQLVPEFDNFLKASAD      278
DsDyP          VILCGREN-------DVDAN-NNNTPFVRPPWALDGSFLALRYLFQLVPEFNTFLTQSAD      278
```

```
                                                 **::.  :    : :   :
                        W264
DyP2          ---------TEQQELVIGRRKLSDVELSDEEKPADSHIALNVITDDEGNELDILR-DNMP    247
AnaPX         K--------LNIQENLAGALIVGRFP-------DGTPVTLSD---IPTYAVTP--TNNFN    316
AauDyP        ----AGSGSLQARADLLGARMVGRWK-------SGAPIDLTPTADDPALGADAQRNNNFT    282
Il-DyP4       Q-NQAGNLTVEEGAELLGSRMFGRWK-------SGAPIDLSPDFDDPALGNDIERNNNFN    344
BadDyP        PANSAGNLTQQEGAEFLGARMFGRWK-------SGAPIDLAPTADDPALGADPQRNNNFD    292
FmDyP         A---DSQVAAENQVELAGARMFGRWK-------SGAPTALSPFADDPALADDPQKNNNFN    308
VvDyP         D---SPGLTKEQGKELLGARLVGRWK-------SGAPVDITPFIDNPQLALDPTRNNNFH    336
Pleos-DyP     V---LPGLSRKEGSELLGARIVGRWK-------SGAPIEITPLKDDPKLAADAQRNNKFD    311
TvDyP1        K---EASLTPEQGSELLGARLMGRWK-------SGAPVDIAPFQDDPVLAADPLRNNNFN    323
ShDyP         K---DSNMPPDQASELLGARMVGRWK-------SGAPIDMTPFEDEPSLGKDPTRNNNFR    317
HiDyP         D---LPGLTRAEGSELLGARFMGRWK-------SGAPIDISPLKDDPALATDPKKNNNFR    323
GlDyP         P---T----KDFTSDLLGARLVGRWK-------SGAPVDLFPLADNPDAGKDPLQNNNFR    324
DsDyP         P---T----TGLSSDLLGARLVGRWK-------SGAPVDVFPLQDNPEAGTDPSQNNNFR    324

                        . *  ..          .:   :                :::
              Y293                                                Y340
DyP2          FGRPGHDEFGT----YFIGYTKS---PAVIEKMLE-------------N---MFVGSPPG    284
AnaPX         YDN-----DLAATKCPFHSHTRKTNPRGDTARLLTADAHFDEAFKEEKGHRITRRAVSYG    371
AauDyP        YSHAGFDLGSDQSHCPFSAHIRKTRPRADLGGSLTPPNLS------AGANSIMRSGIPYG    336
Il-DyP4       YSHPGSDLATDQTRCPFTAHIRKTNPRDLEGQGLFGD-----------TFHAIRAGTPYG    393
BadDyP        YSD----TLTDETRCPFGAHVRKTNPRQDLGG--PVD-----------TFHAMRSSIPYG    335
FmDyP         FSE-V--NLNDQTRCPFAAHVRKSNPRKDLTSVPAFGEAF------VESHMISRQSVPYG    359
VvDyP         FAA----ERDFQKLCPFAAHIRKTLPRADLEASGI----S------LESRRIMRRGIQFG    382
Pleos-DyP     FGDSL--VRGDQTKCPFAAHIRKTYPRNDLEGPPL--KAD------IDNRRIIRRGIQFG    361
TvDyP1        YTAEN--D-NRGANCPLAAHTRKGNPRHDLQDMPI--PIP------LEPHRIIRRGIPFG    372
ShDyP         FE--G--EVDSQDRCPFAAHIRKTNPRADLEDHGT----L------IGKNRIIRRGIQFG    363
HiDyP         FQ--G--EANSQTRCPFSAHVRKTNPRADLEDTKP--PNS------TENRRIIRRGVQFG    371
GlDyP         YDFPD--DFKTQDRCPFAGHTRKTNPRNDLESLG----FS------TENRRIIRRGVQFG    372
DsDyP         YDFPD--DQQTQDRCPFAAHTRKTNPRADLEDLN----IS------TESRRIIRRGVQFG    372

              :               : .: :.                            .    *
                Y347             Y365            W380
DyP2          ---NYDRILDFSTPHTGALFYVPTAD-------F----LEDPPDAPPEPE------PPAA    324
AnaPX         ENNPNK-----EPVLGSGLLFLCFQSNIENQFNFIQSRWANPQNFVQV------------    414
AauDyP        PEVTSAESASNTTTQERGLAFVAYQAQLSQGFHFLQQTWADNANFPPGKTP---------    387
Il-DyP4       PEVTDYEASSNTTTIDRGLAFVEYQSVIGNGFRFQQQAWANNPRFPFSKGPS--------    445
BadDyP        PETSDAELASGVTAQDRGLLFVEYQSIIGNGFRFQQINWANNANFPFSKP----------    385
FmDyP         PEVDPDA-EKFTTVNDRGLAFVCYQSNIDNGFEFVQKTWANDVLFPPLKLNG------IL    412
VvDyP         PELTKQEKRERKTIHGRGLLFVCYQSSIVDAFQFIQKRWSNEPRFPPFERA---------    433
Pleos-DyP     PEVTSQEHHDKKTHHGRGLLFVCYSSSIDDGFHFIQESWANAPNFPVNAVTSAGPIPPLD    421
TvDyP1        PEVTADEAASGKTNQSRGLIFVCYQSNLADGFTFIQKTWANQPLFPLKGLAP-------P    425
ShDyP         PEVTKKEREEGKTIEDRGLIFACYQSNIGNGFKFIQQSWANEPDFPHK------------    411
HiDyP         PELTAEEQHANKTIHGRGLIFAAYQANIANGFQFIQHSWANNQKFIFGTT----------    421
GlDyP         PELTHEEVSSGKTQHGRGLIFVAYCGSITNGFQFIQQSWANNPGFPIQKP----------    422
```

```
DsDyP          PELTPAEISSGKTIEQRGLIFAAYSGSITNGFQFVQHSWADNTGFPINKP----------     422
                                .* :            *      :

                                                                   Y435
DyP2           ATPAGTSLGIGSLR-----RSTRS-------------------------------------     343
AnaPX          --NTGPDPLIGQPS----GTQKWPKKWGEP----ETEEYNFKLWINMKGGEYFFAPSISF     464
AauDyP         -ATVGLDPIIGQNN-----GQPRVVNGLLPSNSSASLSIPQ--FVVSHGGEYFFSPPISA     439
Il-DyP4        -IQLGLDPVIGQ-------GSPRETFGLDPRNASESFTVPQ--VIISNGGEYFFSPSITA     495
BadDyP         -ITPGIEPIIGQ-------TTPRTVGGLDPLNQNETFTVPL--FVIPKGGEYFFLPSISA     435
FmDyP          PLVPGFDPIIGQNN-----GNDRQMAGLQIDHPAQDTKLPIE-FVVSKGGAYFFSPSITA     466
VvDyP          PEEPGFDPIIGQGQ----S--GRKLSGYHPDRPQDELLLPDELFVVPRGGEYFFSPSLKG     487
Pleos-DyP      GVVPGFDAIIGQKV----GGGIRQISGTNPNDPTTNITLPDQDFVVPRGGEYFFSPSITA     477
TvDyP1         VPVPGFDAIIGQAT----DETSRTIAGTDPLNSTGTLHLPTE-WVVPRGGEYFFSPSLPA     480
ShDyP          --GAGLDPIIGQTN----SAGVRHMPGTDPLSQGKDLTIPMQ-FVVSKGGEYFFAPSIST     464
HiDyP          -VEPGFDPIIGQAG----SDGARTVSGSNPKAQANMLTLPTQ-WVVPKGGEYFFSPSIPA     475
GlDyP          -ITPGFDAIIGQNNQGPNGIGPRSMSGANPNNQNATLSLPTE-WVVPKGGEYFFSPSIPA     480
DsDyP          -VTPGFDAIIGQN-----GNGPRSLSGANPKNQSATLALPAN-WVVPKGGEYFFSPSIPA     475
                   * .   **.

DyP2           --------------- 343
AnaPX          LKTLA---------- 469
AauDyP         IGGRLSA-------- 446
Il-DyP4        IVEKFAA-------- 502
BadDyP         LTATIAA-------- 442
FmDyP          LKTKFSV-------- 473
VvDyP          LKEKFTA-------- 494
Pleos-DyP      LKTKFAI-------- 484
TvDyP1         LRSTFAQAS------ 489
ShDyP          LRDVFAVNMHIEAQK 479
HiDyP          LRDTFALGVHIESY- 489
GlDyP          LRDTFSLA------- 488
DsDyP          LRSTFALA------- 483
```

Fig. S1 Sequence alignments of tryptophan and tyrosine residues in *Il*-DyP4 with other representative class V DyPs. Highlighted residues include the following: (i) tryptophan residues were shown in *red* and the corresponding tryptophans in *Il*-DyP4 were indicated in the black box. (ii) tyrosine residues were shown in *green* and the corresponding tyrosines in *Il*-DyP4 were indicated in the black box. Above amino acid sequences of class V DyPs were downloaded from NCBI database: DyP2 from

Amycolatopsis sp. (GI: 496374264), AnaPX from *Anabaena* sp. (GI: 1772287408), *Fm*DyP from *Fomitiporia mediterranea* (GI: 595785908), *Vv*DyP from *Volvariella volvacea* (GenBank: AKU04643.1), *Tv*DyP1 from *Trametes versicolor* (GI: 636616485), *Sh*DyP from *Stereum hirsutum* (GI: 597911761), *Hi*DyP from *Heterobasidion irregulare* (GI: 575067022), *Gl*DyP from *Ganoderma lucidum* (GenBank: ADN05763.1), *Ds*DyP from *Dichomitus squalens* (GI: 1585543766), *Aau*DyP from *Auricularia auricula-judae* (GI: 1048348430) and *Bad*DyP from *Bjerkandera adusta* (GI: 116666995).

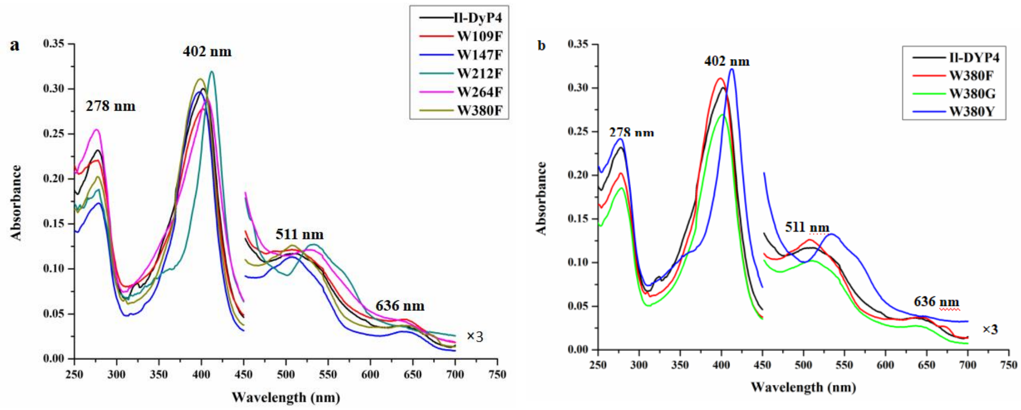

Fig. S2 Electronic absorbance spectra of *Il*-DyP4 and the variants. (a) Absorbance spectra of *Il*-DyP4 and the W variants. (b) Absorbance spectra of *Il*-DyP4 and the W380 variants. All samples used in these assays had about 0.1-0.11 mg/mL of each of enzymes in 10 mM sodium acetate buffer, pH 6.0.

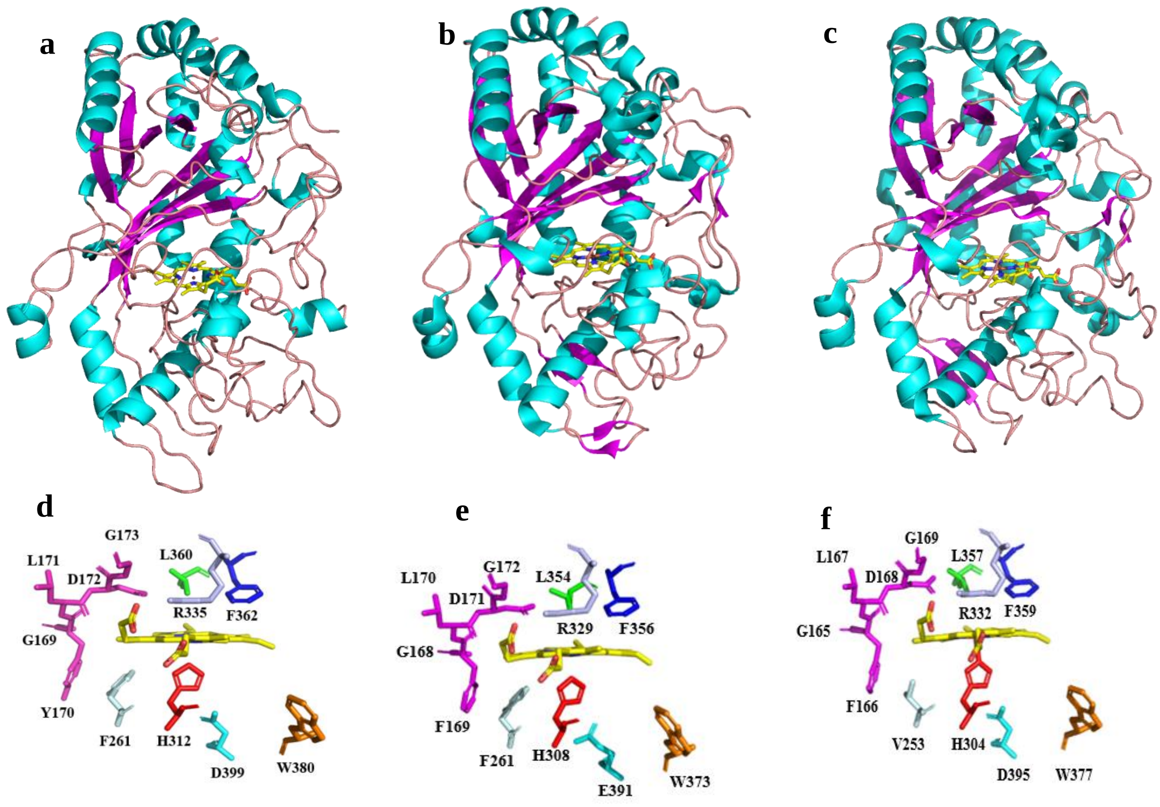

Fig. S3 The structure comparison between *Il*-DyP4 and other fungal DyPs. a-c represent *Il*-DyP4 (PDB: 7D8M), *Bad*DyP from *Bjerkandera adusta* (PDB: 3AFV) and *Aau*DyP from *Auricularia auricula-judae* (PDB: 4W7J), respectively. d-f show the important residues surrounding the heme of each protein and the suggested surface-exposed catalytic tryptophan is showed in orange.

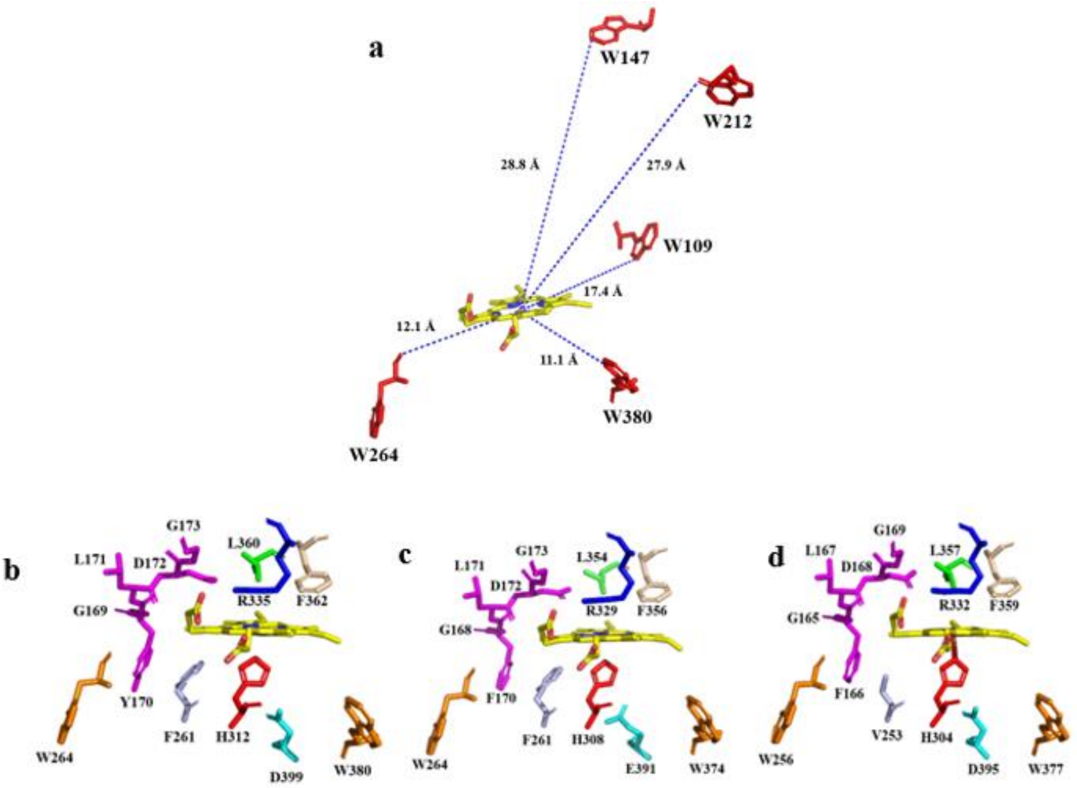

Fig. S4 The location of W264 in *Il*-DyP4 and the comparison with other fungal DyPs. a represents the distances (Å) between the above tryptophan residues and the haem iron in *Il*-DyP4; b-d represent *Il*-DyP4, *Bad*DyP and *Aau*DyP, respectively, showing the position of W264 in *Il*-DyP4 and the corresponding residues in *Bad*DyP and *Aau*DyP.

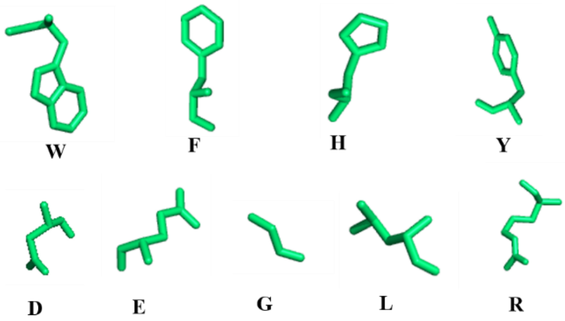

Fig. S5 The structure comparison between W and those amino acids used in W264 mutation.

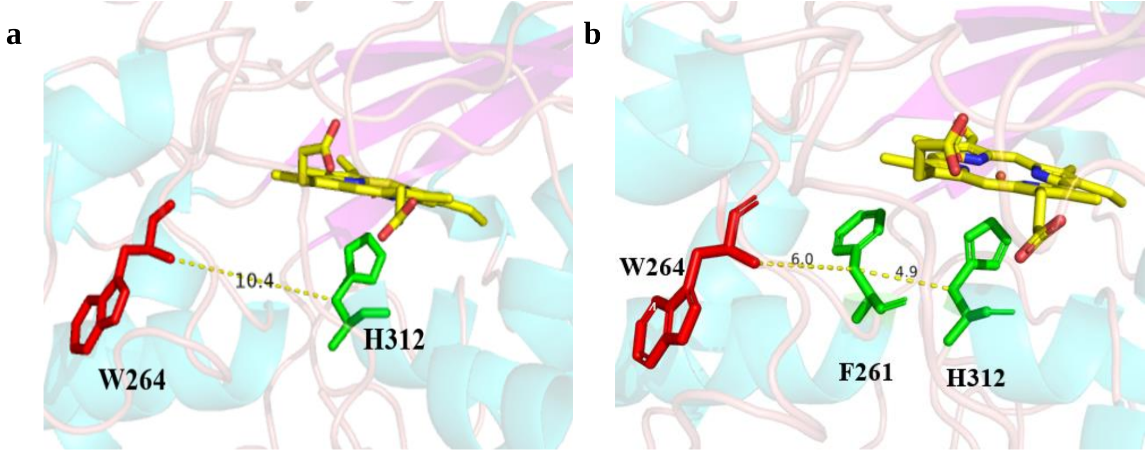

Fig. S6 a shows the distance between W264 and H312. b shows the F261 between W264 and H312. Yellow sticks represent the heme cofactor.

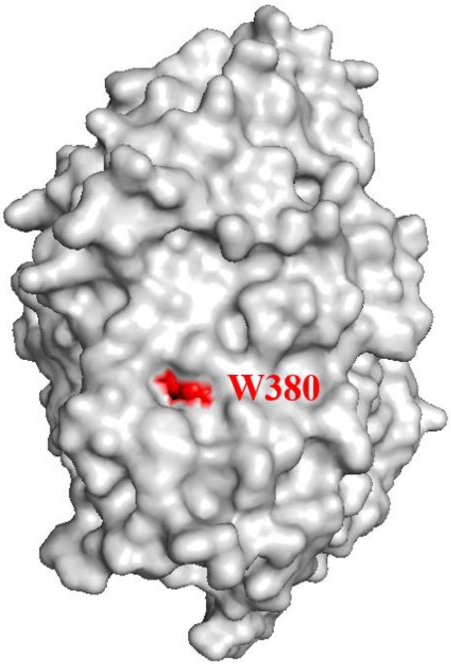

Fig. S7 Location of W380 in *Il*– DyP4. The molecular surface is shown in white. The red sphere represents W380.

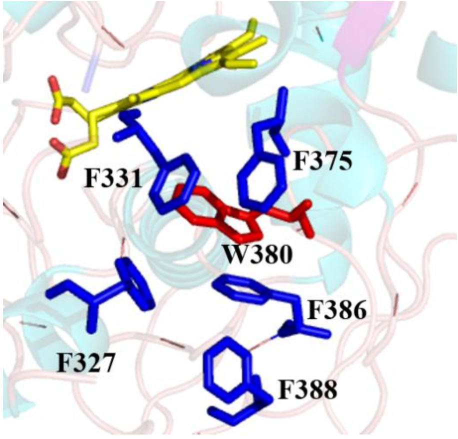

Fig. S8 The hydrophobic phenylalanines surrounding W380 in *Il*- DyP4. The yellow sticks represent the heme.

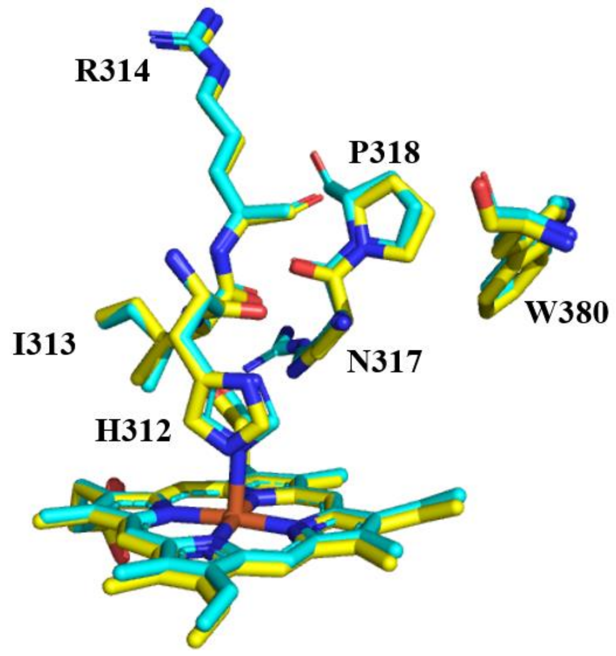

Fig. S9 Structural superpositions of *Il*-DyP4 (yellow, PDB: 7D8M) and *Aau*DyP (azure, PDB: 4W7J) showing a similar electron transfer pathway from the surface-exposed W380 to heme (according to Linde et al. 2015 [27]).
